# Dissecting sexual dimorphism in aortic valve stenosis by proteomics

**DOI:** 10.1186/s12014-025-09549-1

**Published:** 2025-10-06

**Authors:** Ana Grego, Cláudia Sousa-Mendes, Diana Martins, Carla Sousa, Ana Filipa Ferreira, Francisca Saraiva, Inês Alves, Guadalupe Espadas, Isabel Miranda, Adelino Leite-Moreira, Eduard Sabidó, António S. Barros, Cristina Gavina, Rui Vitorino, Inês Falcão-Pires, Rita Nogueira-Ferreira, Fábio Trindade

**Affiliations:** 1 RISE-Health, Department of Surgery and Physiology, Faculty of Medicine, University of Porto, Porto, 4200-319 Portugal; 2 Department of Cardiology, University Hospital Center São João, ULS São João, Porto, 4200-319 Portugal; 3 Proteomics Unit, Center for Genomic Regulation, Barcelona Institute of Science and Technology (BIST), Barcelona, 08003 Spain; 4 Proteomics Unit, Universitat Pompeu Fabra, Barcelona, 08003 Spain; 5 Department of Cardiothoracic Surgery, University Hospital Center São João, ULS São João, Porto, 4200-319 Portugal; 6 Department of Cardiology, Pedro Hispano Hospital, ULS Matosinhos, Matosinhos, 4464-513 Portugal; 7 iBiMED - Institute of Biomedicine, Department of Medical Sciences, University of Aveiro, Aveiro, 3810-193 Portugal; 8 LAQV/REQUIMTE, Department of Chemistry, University of Aveiro, Aveiro, 3810-193 Portugal

**Keywords:** Aortic valve stenosis, Proteomics, Sexual dimorphism, Calcification, Fibrosis, Oxidative stress

## Abstract

**Background:**

The treatment of aortic valve stenosis (AVS) remains limited to aortic valve replacement (AVR). No pharmacotherapy has yet proven efficacious, and its development is challenged by sexual dimorphism. Women display extensive valve fibrosis, and men present remarkably higher valve calcification. To accelerate the development of sex-personalised therapies, deeper molecular insights are needed. Hence, we aimed to characterise AVS sexual dimorphism using proteomics.

**Methods:**

Fifty surgically excised valves (50% women) were homogenised, and the proteins were quantified by LC-MS/MS. The influence of differentially expressed proteins (DEPs) in sexual dimorphism was appraised using bioinformatics. DEPs were validated using immunohistochemistry, qRT-PCR and ELISA, with 30 additional valves.

**Results:**

We quantified ~ 4,000 proteins and 76 DEPs between sexes. CD163, CD74, and NADPH oxidase-2 (NOX2) were more abundant in men’s valves and central in a protein-protein interaction network. Functional enrichment analysis (FEA) supported increased lipoprotein binding and macrophage activation in men’s valves, confirmed by increased CD74 + cell infiltration (immunohistochemistry). Aminopeptidase N, coagulation factor XIII, and metalloreductase STEAP4 were more abundant in men’s valves at the transcript and protein levels. FEA indicated a women-specific dysregulation of spliceosomal proteins that may dictate a pro-fibrotic phenotype, which was observed histologically. A higher glutathione peroxidase-1/NOX2 ratio (ELISA) was found in women, suggesting increased protection against oxidative stress.

**Conclusions:**

Proteomics confirms sexual dimorphism in AVS. Women displayed a higher degree of fibrotic remodelling, whereas men displayed greater immune cell infiltration and were less protected from oxidation, favouring calcification. Proteomics identified putative targets for a sex-personalised AVS modulation.

**Graphical abstract:**

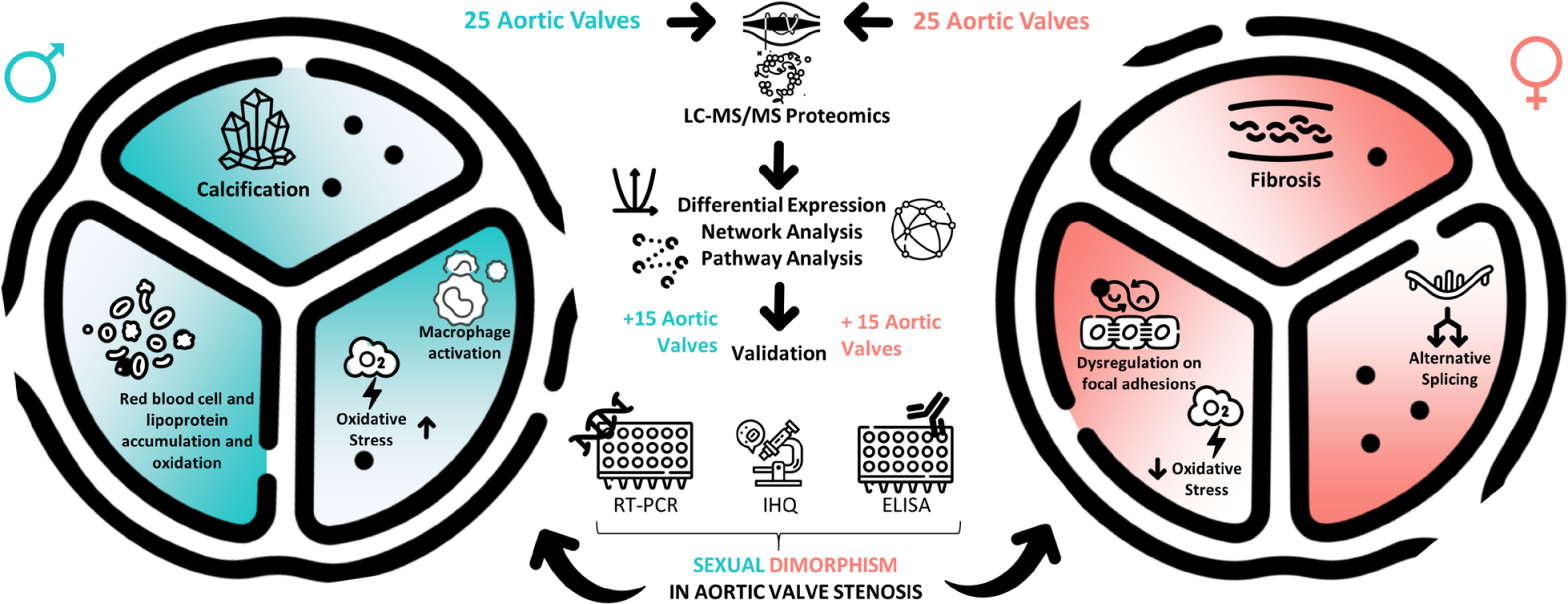
We studied sexual dimorphism in aortic valve stenosis following a proteomic approach. Proteins were quantified by mass spectrometry and sex differences were uncovered by differential expression, network and pathway analyses. Some targets were selected for validation by PCR, immunohistochemistry and ELISA. Collectively, our experiments support a higher propensity for men’s valves to accumulate lipoproteins, red and white blood cells, with concomitantly greater activation of macrophages, particularly CD74^+^. Men’s valves also show a higher propensity for oxidative stress and calcification. Women’s valves are less prone to oxidative stress (higher GPX1/NOX2) but show a greater extent of fibrosis, which might result from an alternative splicing program that translates into a significant dysregulation of focal adhesion proteins. Some graphical elements were retrieved from Flaticon (https://www.flaticon.com/).

**Supplementary Information:**

The online version contains supplementary material available at 10.1186/s12014-025-09549-1.

## Introduction

Aortic valve stenosis (AVS) is the valve disease responsible for most surgeries and catheter interventions in Europe and North America [1]. The main pathological hallmark of AVS is fibrocalcific remodelling and thickening of the valve that limits its maximal opening (stenosis) and thus restricts cardiac blood outflow [2]. The lack of effective pharmacotherapies to revert or delay this phenomenon renders aortic valve replacement (AVR) the only effective treatment to alleviate the left ventricle (LV) afterload. AVR ultimately aims to prevent further myocardial remodelling and progression to heart failure [3].

The initiation of AVS is a complex process influenced by multiple factors. Rheumatic heart disease is still an important cause of AVS in countries of low-to-middle income [4]. In developed countries, degenerative AVS is the most common presentation and can be triggered by mechanical stress, endothelial dysfunction, lipid deposition, and inflammation. For instance, patients with bicuspid valves, a congenital defect that affects up to 2% of the population, leads to increased mechanical stress, which accelerates degeneration at the valve commissures [5]. Although not definitely established as an initiating factor, endothelial dysfunction plays a key role in the early phases of AVS, and is driven by, among others, endothelial nitric oxide synthase uncoupling, which contributes to oxidative stress [6]. The combination of endothelial damage and lipid deposition and subsequent oxidation triggers an inflammatory process within the valve [7]. This is sided by the infiltration of macrophages, T and mast cells, particularly evident in men [8]. This chronic immune-inflammatory process induces the transformation of valve interstitial cells (VICs) into a myofibroblastic phenotype, which is responsible for collagen deposition and fibrotic remodelling. The chronic inflammatory process and the delayed phagocytosis increase the apoptosis rate, with the apoptotic bodies functioning as nucleation sites for microcalcification. Further differentiation of myofibroblasts to the osteogenic phenotype contributes to active calcification. Ultimately, the growth of calcification deposits reduces valve pliability and narrows the aortic valve orifice [6, 9].

Apart from congenital defects such as bicuspid valves, traditional atherosclerosis-related risk factors such as hypertension, dyslipidaemia, obesity, diabetes, metabolic syndrome, smoking and even male sex overlap with AVS [2]. Both atherosclerosis and AVS are characterised by endothelial damage, lipid deposition, inflammation, macrophages and lymphocytes infiltration and neovascularisation. However, disease progression diverges notably. AVS is marked by the involvement of VICs, myofibroblast and osteoblast-like cells, altogether leading to fibrosis and calcification that contribute to progressive valve rigidity and cardiac decompensation. In contrast, atherosclerosis involves foam cells and vascular smooth muscle cells that lead to lipid accumulation and plaque formation that may induce ischaemia or may rupture and induce thrombosis [10]. Given the resemblance to atherosclerotic disease, it was hoped that statins would effectively halt AVS progression. Yet, the attempts to repurpose these drugs have all failed [11–13], showing once more that the progression of these two conditions is shaped by different pathways. Other strategies have reached clinical trials, including drugs targeting proprotein convertase subtilisin/kexin type 9 (PCSK9), lipoprotein(a), matrix Gla protein, soluble guanylate cyclase or dipeptidyl peptidase-4. However, these still need to demonstrate clear haemodynamic benefits in larger randomised controlled trials in both sexes [6].

The development of pharmacological interventions for AVS is complicated by outstanding sexual dimorphism in disease development. A remarkable difference is that women present more valvular fibrosis, and men show more extensive calcification after matching for age, body mass index (BMI), hypertension, renal disease, diabetes mellitus, and the same level of disease severity [14]. This is reflected in sex-specific thresholds for the calcium score, as assessed by computed tomography (CT), to estimate the severity of AVS. Women with > 1,600 or men with > 3,000 Agatston units are very likely to have AVS [1]. In addition, women usually develop milder symptoms, or these become more evident later in the course of the disease, explaining a relative deferral to AVR compared to men. The more advanced age at diagnosis and the risk of more severe complications make these women better candidates for transcatheter valve implantation [15]. Men usually exhibit earlier and more severe symptoms and more frequently have bicuspid valves (more prone to haemodynamic stress) and atherosclerotic vascular disease, explaining why men are referred to surgical AVR more often [16].

Evidence of sexual dimorphism in AVS extends to histological, cellular, and molecular levels. For instance, Simard et al. [14]. reported higher collagen content and a greater fraction of dense connective tissue in women’s valves. Others reported higher amounts of amorphous calcium phosphates in women’s valves and compact hydroxyapatite bone-like deposits in men’s valves [17]. Such histological findings suggest that calcification may be slower in women and governed by different mineralisation pathways, potentially involving different cells and molecular pathways. Indeed, Myasoedova et al. [8]. recently showed a sex-specific enrichment of various cell types in stenotic valves. While women’s valves are enriched in chondrocytes, fibroblasts, osteoblasts, and pericytes, men’s have a significant representation of a myriad of immune cells, including T and B cells, dendritic cells, macrophages, and monocytes. Therefore, women’s overrepresented pathways included mRNA splicing regulation, the regulation of tyrosine kinase receptor, or elastic fibre formation (pro-fibrotic), while men’s included, for instance, innate and adaptive immune responses, interferon and interleukin signalling pathways, apoptosis, reactive oxygen (ROS) and nitrogen species production, collectively sustaining a pro-inflammatory, pro-oxidant and pro-calcific environment [8]. Finer differences at the molecular level are mainly derived from preclinical experiments. For example, the LDL receptor was among the 180 genes differentially expressed between healthy male and female porcine VICs through transcriptomics. This receptor was overexpressed in male cells, suggesting these cells could be more active in lipid management and deposition [18]. Conversely, women’s VICs showed significantly better cholesterol efflux to plasma HDL through ABCA1, a key cholesterol transporter [19]. Another exemplary study shows that women’s VICs can be shielded from interferon-alpha/lipopolysaccharide-induced calcification due to a higher expression of the mineralisation inhibitor matrix-Gla protein and the anti-apoptotic protein Bcl-2 [20].

The evidence of sexual dimorphism in AVS at various levels is growing; however, a comprehensive molecular characterisation of this phenomenon is lacking, particularly at the protein level. Such a characterisation is essential for the development of targeted and sex-specific therapies designed to halt or reverse AVS. To this end, the present study sought to utilise an in-depth proteomic analysis to investigate human-level sexual dimorphism in AVS.

## Materials and methods

### Study population

AVS patients were selected based on a retrospective analysis of clinical data (*N* = 80). Only patients with preoperative echocardiographic confirmation of severe AVS, irrespective of the number of cusps (two or three) and undergoing surgical valve replacement, were included. A severe AVS was defined as (1) a mean transvalvular pressure gradient > 40 mmHg; (2) an indexed aortic valve area < 0.6 cm^2^/m^2^; (3) echocardiographic evidence of relevant leaflet calcification (confirmed in the surgery report) in combination with left ventricle hypertrophy (indexed left ventricle mass ≥ 95 g/m^2^ for women and ≥ 115 g/m^2^ for men) not explained by hypertrophic cardiomyopathies or other conditions. The exclusion criteria included rheumatic AVS, severe aortic valve regurgitation, and lack of diagnostic echocardiography. The protocol was approved by the ethics committee of Centro Hospitalar Universitário de São João (reference CEC109-2020, 20/05/2020). All participants provided written informed consent. The study protocol abided by the principles outlined in the 1964 Declaration of Helsinki and its later amendments.

Clinical and demographic variables, including sex, age, BMI, comorbidities (e.g., hypertension, diabetes mellitus), and medication use, were obtained from medical records. Disease severity was assessed by transthoracic Doppler echocardiography. The mean (taoGmean) and maximal (taoGmax) aortic transvalvular pressure gradients and aortic valve area (AVA) were derived from the modified Bernoulli equation and the standard continuity equation, respectively. Whenever possible, the Doppler velocity index (DVI) was recorded. As an additional measure of disease severity, the left ventricle mass (LVM) was estimated following derivation of left ventricle end-diastolic dimension (LVEDD), posterior wall thickness (PWT), and interventricular septal thickness (IVST) from 2D echocardiograms during diastole with application of Deveraux’s formula (LVM = 0.8 × 1.04 × [(LVEDD + PWT + IVST)^3^– LVEDD^3^] + 0.6 g). Correct orientation of imaging planes, cardiac chambers dimension, and function measurements were performed according to the European Association of Echocardiography/American Society of Echocardiography recommendations [21].

### Sample collection and processing

Aortic valves were obtained as a by-product of surgical AVR, isolated or concomitant with other procedures, in most cases, coronary artery bypass grafting, without any additional risk to the patients. Each sample was immediately immersed in cardioplegic solution (Custodiol^®^) and kept at 4 °C until delivery to the laboratory (< 2 h). Each valve leaflet was washed thrice with phosphate-buffered saline (PBS) to remove residual blood. Samples were weighed to obtain an indirect measure of calcium content. A representative transversal cut was obtained for histology. The remaining material was grossly fragmented and randomly processed for RNA or proteomics/protein analyses. For the former, the fragments were preserved in RNA later™ (Sigma) and incubated overnight at 4 °C before storage at −80 °C. For the latter, the fragments were directly stored at −80 °C until further processing.

### Histology

Aortic valve tissue was automatically processed using a LEICA HistoCore Pearl processor. AV tissue was fixed by diffusion of 4% (V/V) buffered formaldehyde, followed by dehydration with ethanol in crescent concentrations, clearing with xylene, and paraffin impregnation. Paraffin-embedded samples were then included in blocks in the LEICA HistoCore Arcadia. After refrigeration, the paraffin blocks were cut into 3 μm-thick sections using a microtome (LEICA RM2125 RTS). The top sections were discarded, and when the sample emerged, it was subjected to gross decalcification by incubation in a decalcifying solution with ethylenediamine tetraacetic acid (Osteosens, Biognost^®^, Croatia) for 5 min. Then, sample sections were cut and placed on sequential water baths, first at room temperature (RT) and then at 40 °C, to unfold the tissue slices. Finally, the sections were allowed to dry on glass slides.

Before staining, slides were deparaffinised and rehydrated. First, tissue paraffin sections were heated at 60 °C for 30 min to dissolve paraffin. The slides were then deparaffinised twice with fresh xylene (10 min + 5 min) and dehydrated with decreasing ethanol concentrations (100%, 80%, and 70%, 2 min each). Finally, the slides were rinsed with tap water for 5 min for hydration. All staining procedures were performed at RT.

Slides were stained with Haematoxylin and Eosin (H&E) to evaluate the general morphology and tissue architecture. First, slides were placed in modified Harris haematoxylin (Biognost^®^) for 8 min and then washed with tap water. The slides were briefly immersed in acid alcohol (hydrochloric acid 37% in 70% ethanol, 1:100) and washed with tap water between the stains. Next, the slides were placed in alcoholic eosin Y 1% (0.2–0.5% acetic acid, 70–90% ethanol, Biognost^®^) for 2 min. Finally, the preparations were dehydrated with ethanol (90% for 30 s and 99.5% for 1 min) and cleared with xylene for 5 min.

To assess collagen deposition in the aortic valve, the slides were stained with Red Sirius 0.1% (Direct Red 80, in 1.3% picric acid solution, Sigma) for 90 min and then briefly rinsed in acid water (0.5% glacial acetic acid). Next, the slides were dehydrated in three ethanol solutions of 99.5% and deparaffinised twice with xylene for 5 min.

To evaluate the extent of calcification, the slides were placed in 2% Alizarin Red S solution (Sigma) for 5 min and briefly rinsed in water. Next, the slides were sequentially treated with acetone for 1 min and acetone: xylene (1:1) for 1 min. Finally, the slides were cleared twice with xylene for 5 min.

For visualisation and image acquisition, the slides were mounted with Entellan^®^ and observed under an optical microscope (Zeiss Axio Scope.A1, Germany) equipped with a photographic camera (Olympus XC30, Tokyo, Japan). For H&E staining, photographs were taken under 25× magnification, while for Red Sirius and Alizarin Red S staining, a 40× magnification was used. Fibrosis and calcific load were analysed using Image Pro Plus software by an observer and then reviewed by an expert. To minimise potential biases and ensure a standardised and impartial assessment of fibrosis and calcification, the observer and the expert were blind to the patient’s sex until all sections were analysed. This analysis involved calculating the tissue area in each region-of-interest, followed by assessment of the stained areas, divided by the tissue area. Eight regions-of-interest were evaluated for each patient, and the results were averaged. Fibrotic and calcific areas were expressed relative to the total tissue area (%).

### Proteomics

#### Protein digestion and sample clean-up

Fifty AV samples (25 men) participants were selected for proteomic analysis, ensuring matching based on key clinical covariates [22]. Fifty µg of protein of each sample was precipitated with 6 volumes of cold acetone, and the pellet was dissolved in 50 µL of 6 M urea and 200 mM ammonium bicarbonate. Then, samples were reduced with dithiotreitol (150 nmol, 37 °C, 60 min) and alkylated in the dark with iodoacetamide (300 nmol, 25 °C, 30 min). The resulting protein extract was first diluted to 2 M urea with 200 mM ammonium bicarbonate for digestion with endoproteinase LysC (1:100 w/w, 37 °C, over 6 h, Wako, cat #129–02541) and then diluted 2-fold with 200 mM ammonium bicarbonate for trypsin digestion (1:100 w/w, 37 °C, over 6 h, Promega, cat #V5113). After digestion, the peptide mix was acidified with formic acid and desalted with a MicroSpin C18 column (The Nest Group, Inc.) before LC-MS/MS analysis.

#### Tissue homogenisation and protein extraction

Fifty aortic valves were homogenised following a standard operating procedure we previously optimised for the proteomic characterisation of calcified valves [22]. Briefly, the tissue was disrupted with zirconium oxide beads (2.8 mm, Precellys^®^, Bertin Technologies) using a bead-beating system (Minilys, Bertin Instruments). Approximately 30 mg of tissue was combined with ~ 1.2 g of beads in O-ring tubes. Ice-cold RIPA buffer (25 mM Tris-HCl pH 7.6, 150 mM NaCl, 1% NP-40, 1% sodium deoxycholate, 0.1% SDS, Thermo Fisher), supplemented with 1 mM EDTA, a protease inhibitor cocktail (Halt PIC, Thermo Fisher), and a phosphatase inhibitor cocktail (PhosSTOP, Roche) was added at a 10 µL/mg ratio. Then, the samples were homogenised twice, at maximum speed (5,000 rpm) for 30 s, in the Minilys instrument, with a 5-min cool down on ice between cycles. The sample was centrifuged at 12,000 rpm (13,680 × *g*) for 15 min at 4 °C to obtain a clear supernatant. The sample was aliquoted in 100µL fractions and stored at −80 °C until further analysis.

Protein concentration was estimated using the detergent-compatible (DC) method (Bio-Rad Laboratories), following the manufacturer’s instructions. Bovine serum albumin was used as a standard. All samples and standards were prepared in duplicate, and quadruplicate absorbance readings were averaged to obtain the concentration of each sample.

#### LC-MS/MS analysis

Samples were analysed using an Orbitrap Eclipse mass spectrometer (Thermo Fisher Scientific) coupled to an EASY-nLC 1200 (Thermo Fisher Scientific). Peptides were loaded directly onto the analytical column and separated by reversed-phase chromatography using a 50-cm column with an inner diameter of 75 μm, packed with 2 μm C18 particles (Thermo Fisher Scientific, cat #ES903).

Chromatographic gradients started at 95% buffer A (0.1% formic acid in water) and 5% buffer B (0.1% formic acid in 80% acetonitrile) with a flow rate of 300 nL/min and gradually increased to 25% buffer B and 75% A in 105 min and then to 40% buffer B and 60% A in 15 min. After each analysis, the column was washed for 10 min with 100% buffer B.

The mass spectrometer was operated in positive ionisation mode with nanospray voltage set at 2.4 kV and source temperature at 305 °C. The instrument was operated in data-independent acquisition (DIA) mode, with full MS scans over a mass range of *m/z* 500–900 with detection in the Orbitrap at a resolution of 120,000. The auto gain control (AGC) was set to 1e6, and a maximum injection time of 246 ms was used. In each cycle of DIA analysis, following each survey scan, 40 consecutive windows of 10 Da each were used to isolate and fragment all precursor ions from 500 to 900 *m/z*. A normalised collision energy of 28% was used for higher-energy collisional dissociation (HCD) fragmentation. MS2 scan range was set from 350 to 1850 *m/z* with detection in the Orbitrap at a resolution of 30,000. The AGC was set to 1e6, and a maximum injection time of 54 ms was used.

Digested bovine serum albumin (New England Biolabs, cat #P8108S) was analysed between each sample to avoid sample carryover and ensure the instrument’s stability. Qcloud was used to control instrument longitudinal performance during the project [23].

#### Protein identification and quantification

Acquired spectra were analysed using a library-free strategy with DIA-NN (v.1.7.12) [24]. A Swiss-Prot human database (January 2021, 20395 entries) plus a list of common contaminants [25] was used as a reference proteome. For peptide identification, trypsin was chosen as the enzyme and up to one missed cleavage was allowed. Methionine oxidation was used as variable modification, whereas carbamidomethylation of cysteine was set as fixed modification. A false discovery rate (FDR) was set to a maximum of 1% in peptides. Precursor and fragment ion *m/z* mass range were adjusted to 500–900 and 350–1850, respectively. Default settings were used for the other parameters (the full search report is provided as part of Supplementary File 1). Proteins were deemed identified when passing a 1% FDR threshold and those that, in addition, were quantified in at least 75% of the samples of one sex were regarded as confidently quantified. Given the approach used (DIA), no further filter was applied to the number of peptides sequenced per protein.

The raw proteomics data have been deposited to the PRIDE [26] repository with the dataset identifier PXD051201.

### Bioinformatics

#### Functional enrichment analysis

The clusterProfiler package was used to map gene ontology terms pertaining to Biological Processes (BP), Molecular Functions (MF), and Cellular Components (CC) to the DEPs. The human library was obtained on 16 March 2022, and only terms surpassing a 5% FDR were considered.

In addition, cell-type enrichment analysis of the DEPs was conducted using the Enrichr webtool (https://maayanlab.cloud/Enrichr/) to ascertain which cell types were mainly enriched in the valves of men and women. Proteins were mapped to the Human Gene Atlas database, and only cell types with an adjusted *p*-value < 0.05 were considered.

#### Protein-Protein interaction analysis

To identify the most influential proteins in the definition of sexual dimorphism in AVS, a protein-protein interaction (PPI) analysis was performed using the STRING application (v.11.5), available for Cytoscape (v.3.9.1). Only the DEPs were considered for this analysis, and all known interactions with a medium confidence score of 0.4 (default) were retrieved. Topological network analysis was run using Cytoscape built-in tools to calculate node degree and betweenness centrality and identify protein hubs and bottlenecks.

#### Identification of proteins differentially expressed between sexes

Protein data were analysed in R (version 4.2.3) with the package DEP (Differential Enrichment Analysis of Proteomics Data) to identify differentially expressed proteins. A variance-stabilising normalisation method was applied to the protein quantification raw data [27]. Given that the DIA method results in fewer missing values, no data imputation was performed. Missing values were random (no sex-defined cluster was observed) and corresponded to proteins close to the detection limit (Supplementary Figs. 1 and 2). A differential enrichment test based on protein-wise linear models and empirical Bayes statistics using limma was applied to prioritise the most significantly changed proteins, considering an adjusted *p*-value of ≤ 0.2. Only unambiguously identified proteins were considered as DEPs. Protein groups assigned to more than one UniProt ID were removed. The standardised mean difference (Cohen’s d) was used as an additional criterion to select the most relevant proteins explaining sexual dimorphism. DEPs were considered only when they showed a large effect size (Cohen’s d > 0.8).

### RNA extraction and qRT-PCR

RNA was extracted using the Aurum™ Total RNA Fatty and Fibrous Tissue Kit (catalogue #732–6830, Bio-Rad), following the manufacturer’s instructions. This kit was chosen due to its superior performance concerning the extraction yield and RNA quality in calcified valve tissue [28].

RNA was extracted from approximately 100 mg of tissue using 2.8 mm zirconium oxide beads in the same instrument used for protein extraction (Minilys). 1 mL of PureZOL was added to each sample, tissue disruption was performed at maximum speed (5,000 rpm) in 3 cycles of 30 s, and the sample was incubated on ice between cycles. The collected lysate was incubated at RT for 5 min and centrifuged at 12,000 × *g* for 10 min at 4 °C to remove insoluble material. The supernatant was transferred to a new microtube and vigorously mixed with 0.2 mL of chloroform for 15 s. The samples were incubated for 5 min at RT, periodically mixed, and centrifuged at 12,000 × *g* for 15 min at 4 °C to separate the phases. The RNA contained in the aqueous phase was immediately transferred to a new tube and thoroughly mixed with 400 µL 70% ethanol by pipetting. The samples were transferred to an RNA-binding mini-column and centrifuged at 12,000 × g for 1 min at RT to purify the RNA. A low-stringency wash solution (700 µL) was added to the RNA-binding mini-column, and the samples were centrifuged again for 30 s at RT. Each column was incubated with 80 µL of diluted DNase I for 15 min to remove contaminating genomic DNA. Next, 700 µL of high-stringency wash solution was added to the RNA-binding mini-column, and centrifugation was performed at 12,000 × *g* for 30 s at RT. A final wash with 700 µL of low-stringency solution was completed, and the samples were centrifuged twice at 12,000 × *g* for 2 min at RT. To elute RNA, the mini-columns were incubated twice with 25 µL of elution solution for 1 min and centrifuged at 12,000 × *g* for 2 min at RT. The purified RNA was stored at −20 °C until further use.

RNA concentration and purity were measured using the optical density at 260/280 nm and 260/230 nm in a NanoDrop spectrophotometer (Thermo Scientific). RNA integrity was verified using electrophoresis.

From an initial set of 50 samples, RNA was successfully extracted from 47 (24 men and 23 women). Eleven dysregulated proteins were selected for validation at the transcript level, including *AIF1*,* ANPEP*,* CD163*,* CD74*,* DNAJA1*,* F13A1*,* GPX1*,* NOX2* (or *CYBB*), *OSBP1*, *PFKL*, and *STEAP4*, in addition to a panel of 7 genes (*COL1A1*,* COL3A1*,* MMP2*,* MMP9*,* TGFB*,* TIMP1*, and *TIMP2*) associated with fibrosis regulation.

After RNA extraction, cDNA (100 ng/µL) was synthesised using a SensiFAST^™^ cDNA Synthesis Kit (Bioline). A mastermix of the RNA sample, 5x TransAmp buffer, reverse transcriptase enzyme, and DNase/RNase free water was prepared and transferred to the Bio-Rad T100^™^ Thermal Cycler. Reactions were controlled using the following protocol: 10 min at 25 °C for primer annealing, 15 min at 42 °C for reverse transcription, and 5 min at 85 °C to inactivate reverse transcriptase.

Quantitative polymerase chain reaction (qPCR) reactions were run in duplicate in a 10 µL reaction volume, including 1 µL of cDNA, 5 µL 2×SensiFAST™SYBR Hi-ROX Mix (Bioline), and 0.4 µL of primers (Supplementary Table 1). The RT-qPCR reactions were monitored with the PikoReal^™^ 96 and QuantStudio^™^ 5, Real-Time PCR Systems (Thermo Scientific^™^), using the following protocol: 3 min at 95 °C for polymerase activation, followed by 40 cycles of 15 s at 95 °C for denaturation, 30 s at 60 °C for annealing, and 30 s at 72 °C for chain extension. Melting curve analysis was performed from 65 to 95 °C in 0.5 °C increments.

Before quantification of gene expression, the PCR efficiency of each gene, including the internal control gene (18 S RNA), was determined, and it was confirmed that they were identical.

The target gene expression was normalised to 18 S rRNA gene expression. Gene expression was analysed using a comparative method.

### Immunohistochemistry

The differential expression of CD74 and CD163, markers of immune cells, in AV tissue, was validated by immunohistochemistry in 12 patients (6 women). Tissue sections were placed in an antigen retrieval solution (0.1 M citrate buffer, composed of 0.0825 M sodium citrate dihydrate and 0.0175 M citric acid, pH 6.0) and heated in a microwave for 20 min. The slides were allowed to cool to RT, and the tissue sections were outlined with a hydrophobic pen (IHC PAP pen, Enzo). The sections were then incubated in 0.3% H_2_O_2_ (UltraVision hydrogen peroxide block, Thermo Scientific) for 10 min to quench the endogenous peroxidase activity. The slides were washed with PBS, incubated for 5 min with UltraVision protein blocking solution (Thermo Scientific), and washed again with PBS. Next, the tissue sections were incubated with the respective unlabelled primary antibodies, mouse monoclonal anti-CD74 (1:250 in PBS, sc-6262, Santa Cruz Biotechnology) and mouse monoclonal anti-CD163 (1:200 in PBS, sc-20066, Santa Cruz Biotechnology) for 30 min at RT, and washed with PBS. The slides were then incubated with a biotinylated secondary antibody (UltraVision Primary Antibody Amplifier Quanto, Thermo Scientific) for 10 min at RT and washed with PBS. To enhance the signal, sections were incubated with streptavidin-horseradish peroxidase conjugate (UltraVision HRP Polymer Quanto, Thermo Scientific) for 15 min at RT. The slides were washed thoroughly with deionised water and incubated with the corresponding substrate solution (3,3′-Diaminobenzidine, DAB, Thermo Scientific) for 5 min. Haematoxylin (Thermo Scientific) was used as the counterstain. Finally, the sections were dehydrated with ethanol, cleared with xylol, and mounted using Entellan^®^. The primary antibody was replaced with PBS as a negative control.

Slides were photographed under the same microscope under 200× magnification. The protein signal was analysed using the Image Pro Plus software and expressed as the number of DAB spots relative to the total tissue area.

### ELISA

Differences in the protein levels of GPX1 (ref. A78193) and NOX2 (ref. A78533) between sexes were further confirmed by enzyme-linked immunosorbent assay (ELISA), following the manufacturer’s instructions (Antibodies.com, United Kingdom). AV lysates prepared for proteomics and 30 extra samples (*N* = 80) were diluted 10× and 2× with sample dilution buffer (provided by the kit) for GPX1 and NOX2 quantification, respectively. The absorbance of the standards and samples was recorded at a wavelength of 450 nm. Protein concentration was interpolated from the best-fitting curve (sigmoidal in both cases).

### Statistical analysis

Clinical and demographic data was analysed in R (version 4.3.1), and the differences between men and women were queried with the package gtsummary (version 1.7.2). Continuous variables are presented as medians (interquartile ranges), and categorical data as absolute and relative frequencies. The proteome was also analysed in R (see above). To analyse the histological and specific molecular data, we used GraphPad Prism 9.5.1. Differences between sexes in fibrosis, calcification, and immunohistochemistry staining were inspected by an unpaired Mann-Whitney U test. Gene expression data (qRT-PCR) were normalised to RNA18SN1, and protein quantification data (ELISA) were normalised to total protein. Outliers in the PCR and ELISA data were removed using the ROUT method, with the highest level of stringency (Q = 0.1%). Differences in gene expression and protein levels were investigated using unpaired t-tests. Results were considered significant at *p* < 0.05.

## Results

### Characteristics of the study population

Aiming to study sexual dimorphism in AVS at the protein level, we started by characterising the aortic valve proteome in a cohort of 50 patients (25 men and 25 women; Table 1). The patient’s age ranged between 57 and 84 years old at the time of AVR, and 78% were overweight (BMI > 25 g/m^2^). Arterial hypertension, followed by dyslipidaemia, diabetes mellitus and coronary artery disease, were the most frequent comorbidities, with no differences between the sexes. However, past or current smoking habits were more common in men (*p* < 0.05). Additionally, no differences were observed in the prescribed medication between groups. The severity of AVS, as documented by echocardiography, was similar between the sexes regarding transvalvular pressure gradients, indexed aortic valve area, DVI, and even the degree of LV hypertrophy. Men and women presented comparable susceptibility to mild-to-moderate aortic regurgitation (ca. two-thirds). No patients with severe regurgitation were included to avoid bias. Regarding the validation populations (Supplementary Tables 2 and 3), no differences between sexes were observed in the main parameters concerning disease severity, and only punctual differences in prescribed medication for PCR and BMI for PCR and ELISA were observed. Still, the proportion of overweight patients was similar across subpopulations (overweight men: 76%, 87%, 78%; and overweight women: 80%, 96%, 85%, in the proteomic, PCR, and ELISA cohorts, respectively). Therefore, we assume that adding independent samples did not introduce any significant bias to this study.

**Table 1:** Characteristics of the proteomics cohort

	*N*	men, *N* = 25^a^	women, *N* = 25^a^	*p*-value^b^
Age at AVR	50	70 (65,76)	75 (67,77)	0.3
BMI (kg/m^2^)	50	27.0 (25.5,29.4)	27.6 (26.6,31.5)	0.2
*Comorbidities*				
Dyslipidaemia	50	16 (64%)	19 (76%)	0.4
Smoking habits	50	9 (36%)	2 (8.0%)	0.017
Arterial hypertension	50	21 (84%)	19 (76%)	0.5
Diabetes mellitus	50	11 (44%)	8 (32%)	0.4
CAD	50	5 (20%)	2 (8.0%)	0.4
AR (mild-moderate)	50	16 (64%)	17 (68%)	0.8
*Pharmacology*				
Statins	50	17 (68%)	18 (72%)	0.8
Beta-blockers	50	9 (36%)	9 (36%)	> 0.9
ACEi	50	5 (20%)	10 (40%)	0.12
AT2Ri	50	13 (52%)	7 (28%)	0.083
Diuretics	50	14 (56%)	14 (56%)	> 0.9
Anticoagulants	50	1 (4.0%)	2 (8.0%)	> 0.9
Aspirin	50	14 (56%)	9 (36%)	0.2
CAC blockers	50	10 (40%)	6 (24%)	0.2
SGLT2i	50	4 (16%)	0 (0%)	0.11
*Disease severity*				
taoGmax (mmHg)	50	74 (66,82)	80 (65,89)	0.4
taoGmean (mmHg)	50	46 (41,51)	50 (41,60)	0.2
AVAi (cm^2^/m^2^)	50	0.46 (0.41,0.53)	0.46 (0.41,0.50)	0.8
DVI	33	0.22 (0.20,0.26)	0.24 (0.20,0.26)	0.7
LVMi (g/m^2^)	47	127 (114,143)	120 (109,143)	0.8
Bicuspid valves	46	10 (42%)	8 (36%)	0.8

^a^n (%); Median (IQR); ^b^Wilcoxon rank sum test; Pearson’s Chi-squared test; Fisher’s exact test;

ACEi– angiotensin-converting enzyme inhibitors; AR– aortic regurgitation; AT2Ri– angiotensin II receptor inhibitors; AVAi– aortic valve area indexed to body surface area; AVR– aortic valve replacement; BMI– body mass index; CAC blockers– calcium channel blockers; CAD– coronary artery disease; DVI– Doppler velocity index (velocity ratio); LVMi– left ventricle mass indexed to body surface area; SGLT2i– sodium-glucose co-transporter 2 inhibitors; taoGmax– maximal transvalvular aortic pressure gradient; taoGmean– mean transvalvular aortic pressure gradient

**Fig. 1:**
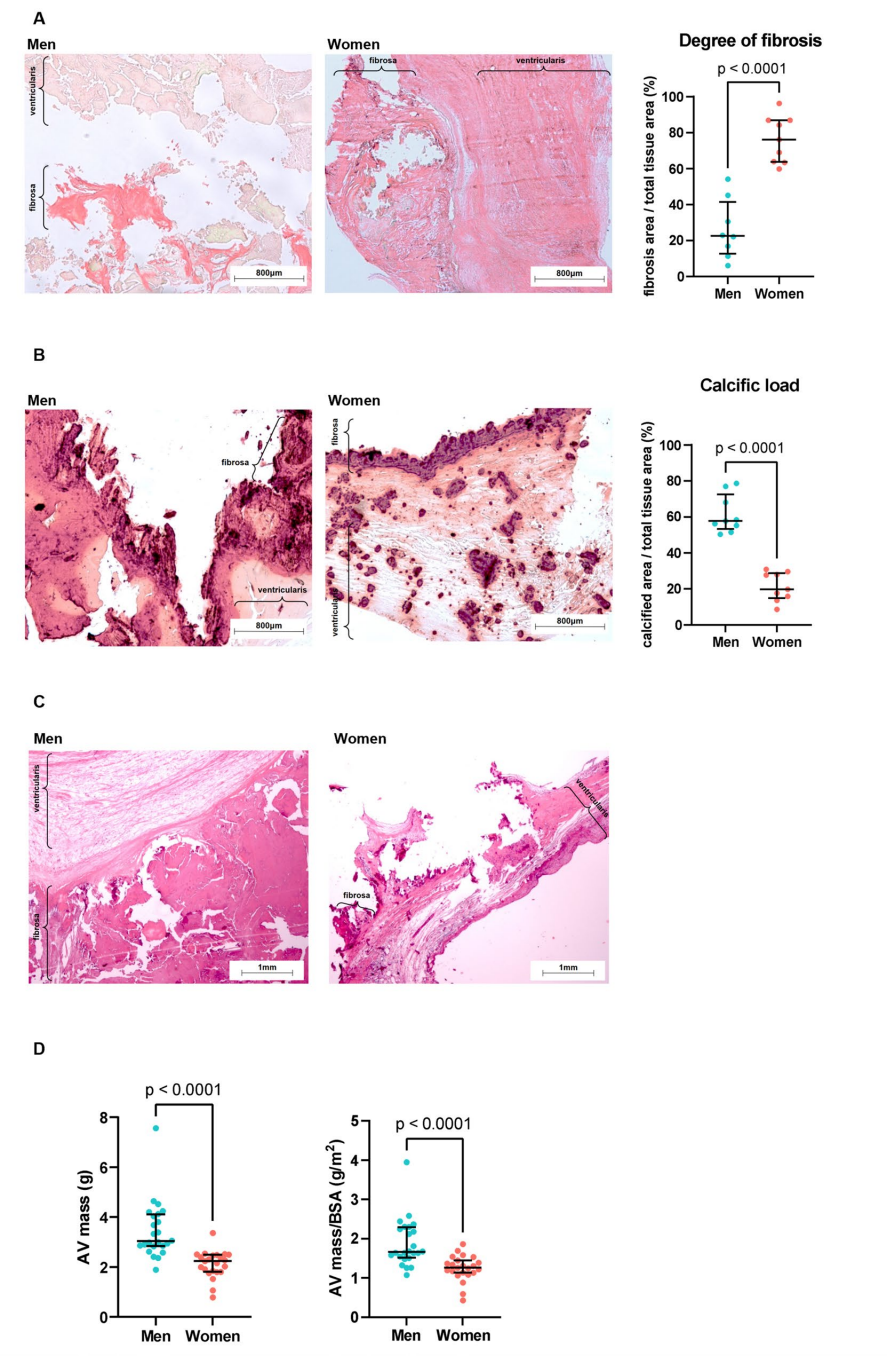
Histological evidence of sexual dimorphism in aortic valve stenosis. (**A**) Representative aortic valve cross-sections (4x magnification) from men and women with stenosis, stained with Sirius Red, and quantification of fibrotic tissue as the % of total tissue area (*n* = 8 men, *n* = 9 women). (**B**) Representative cross-sections (4x magnification) from men and women with stenosis, stained with Alizarin Red S, and quantification of calcified tissue as the % of total tissue area (*n* = 9 men, *n* = 9 women). (**C**) Representative cross-sections (2.5x magnification), stained with haematoxylin and eosin, showing increased valve thickness in a man’s valve. (**D**) Comparison of the excised valve weight between men (*n* = 24) and women (*n* = 23) undergoing surgical valve replacement, showing increased weight in men, even when corrected to body surface area (right). In all cases, data are presented as median and interquartile range and individual biological replicates as dots. Differences were inquired with a Mann-Whitney test. AV, aortic valve; BSA, body surface area

Gross macroscopic observation and histology confirmed the existence of sexual dimorphism in AVS. For the same haemodynamic severity of AVS, women’s valves presented a greater extent of fibrotic areas (76% vs. 26% in men, *p* < 0.0001; Fig. 1A). In comparison, men’s valves were richer in calcium deposits (61% vs. 21% in women, *p* < 0.0001; Fig. 1B), resulting in thicker valves, as evidenced by H&E staining (Fig. 1C). The greater calcium load in men’s valve tissue was reflected in the weight of the excised valves, with men’s specimens being significantly heavier (median 3.0 vs. 2.2 g in women, *p* < 0.0001), even when corrected for body surface area (median 1.7 vs. 1.3 g/cm^2^, *p* < 0.001, Fig. 1D) and aortic valve area (*p* < 0.01, Supplementary Fig. 3).

### Proteome analysis

**Fig. 2:**
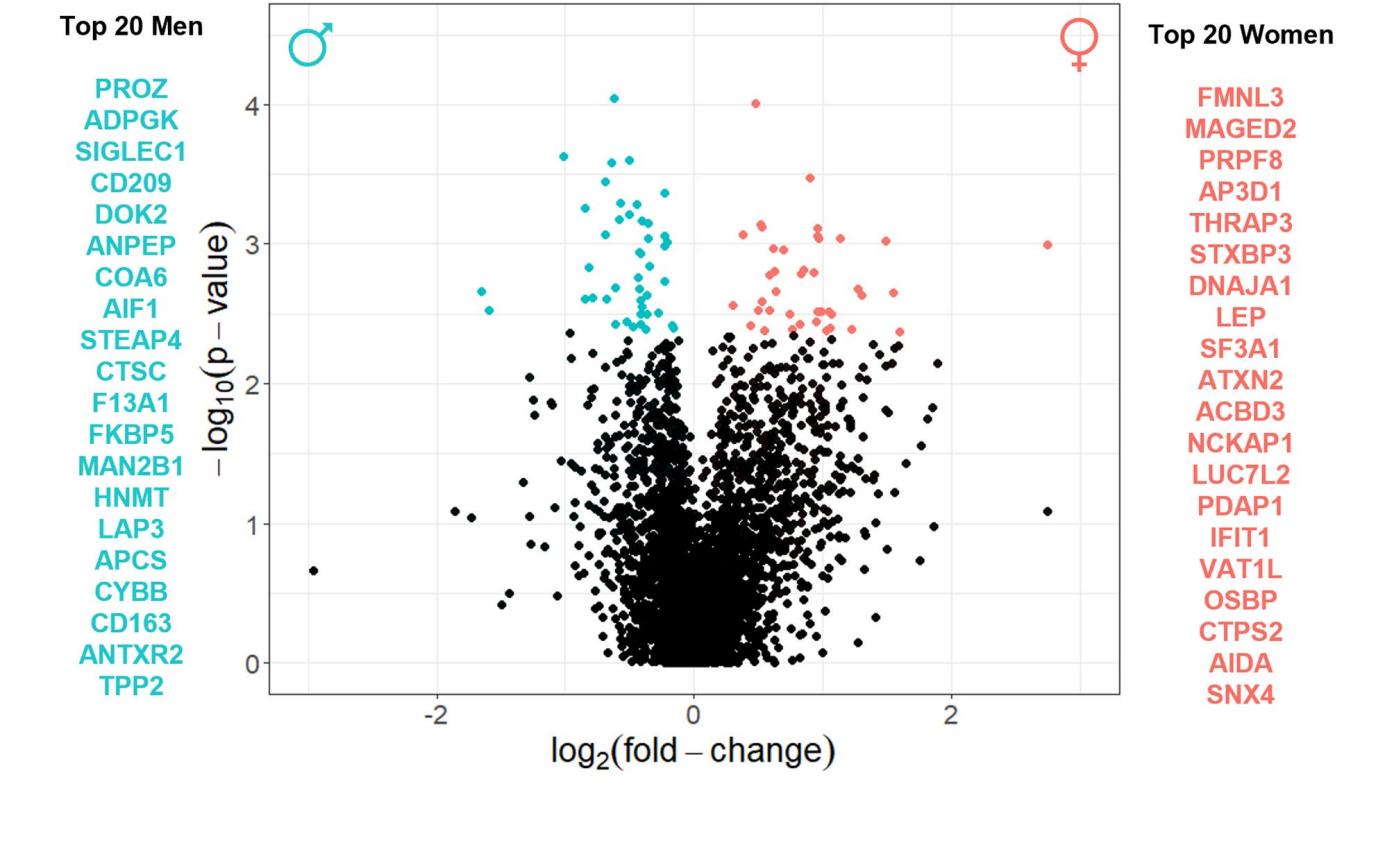
Proteomic characterisation of the sexual dimorphism in aortic valve stenosis. Volcano plot representing the proteins most significantly changed between sexes (*N* = 50; *n* = 25 men, *n* = 25 women), according to a differential enrichment test based on protein-wise linear models and empirical Bayes statistics using limma. Coloured dots represent significantly changed proteins (*p* < 0.05; adjusted p-value ≤ 0.2): proteins upregulated in men are shown in turquoise, and proteins upregulated in women are shown in salmon. For each sex, the top 20 proteins, identified by the respective gene name, are listed on each side of the Volcano

After confirming the sexual differences at the histological level, we aimed to take a holistic view of this dimorphism at the molecular level using proteomics. We analysed the proteome of 50 valves following a DIA strategy with a state-of-the-art quantification method employing neural networks (DIA-NN) for improved proteome coverage [24]. With this approach, we sequenced > 75,000 peptides and initially identified 8,265 proteins (Supplementary File 1). After filtering for proteins that were missing in > 25% in both sexes, we quantified exactly 3,980 proteins (Supplementary File 1), enabling an unparalleled in-depth characterisation of the aortic valve proteome. 84 protein groups were deemed differentially expressed between men and women (Fig. 2), all with a large effect size, from which 76 single proteins (Supplementary Table 4) were considered for downstream analyses. The top 20 proteins with the highest fold-change in expression are listed in Fig. 2.

**Fig. 3:**
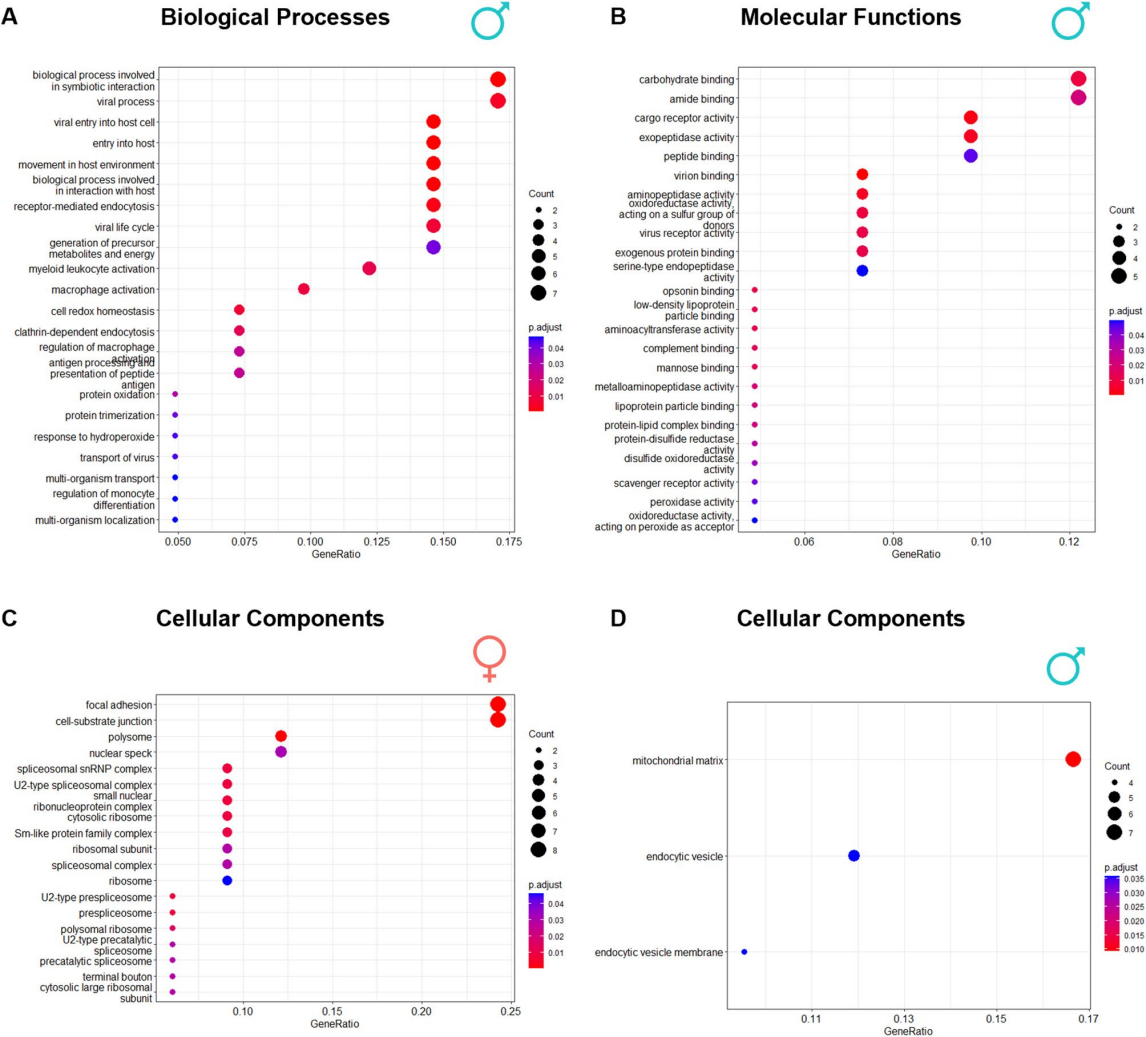
Gene ontology enrichment analysis. Proteins upregulated in men and women were mapped to (**A**) Biological Processes (men only), (**B**) Molecular Functions (men only) and (**C**) and (**D**) Cellular Components (women and men, respectively). No significantly enriched biological processes or molecular function terms were retrieved for women. The dot size is proportional to the number of mapped genes, and its colour represents the significance (the redder, the more significant the enrichment). Analysis was based on the quantification data from all biological replicates (*N* = 50). Enriched terms were obtained through a hypergeometric test, considering an FDR of 5%

FEA was performed to gain insight into the biological pathways distinctly affected in men and women with AVS. Mapping of biological processes showed activation of many immune system-related pathways in men, including leukocyte activation or, more specifically, macrophage activation (Fig. 3A). Indeed, cell type enrichment analysis showed a significant enrichment of CD33^+^ myeloid lineage cells and an even greater enrichment of CD14^+^ monocytes/macrophages in men’s but not in women’s valves (Supplementary Table 5). Prominent enrichment of molecular functions, such as binding to low-density lipoprotein particles, peroxidase activity or complement binding, was found in men (Fig. 3B), corroborating a higher burden of lipoprotein accumulation in the AV, along with intricately associated oxidative stress and complement system-mediated inflammation. No biological processes or molecular functions were significantly enriched in women; however, mapping of cellular components showed prominent enrichment of proteins localised on focal adhesions or cell-substrate junctions, suggesting tissue reorganisation. In addition, enrichment of proteins associated with the spliceosome was observed in women, suggesting women-specific phenotypic differentiation of valve cells (Fig. 3C). In men, upregulated proteins were mainly limited to the mitochondrial matrix (remarkably, oxidative stress-associated proteins) and endosomes, the latter suggesting increased internalisation of oxidised lipoproteins and cell debris (Fig. 3D).

**Fig. 4:**
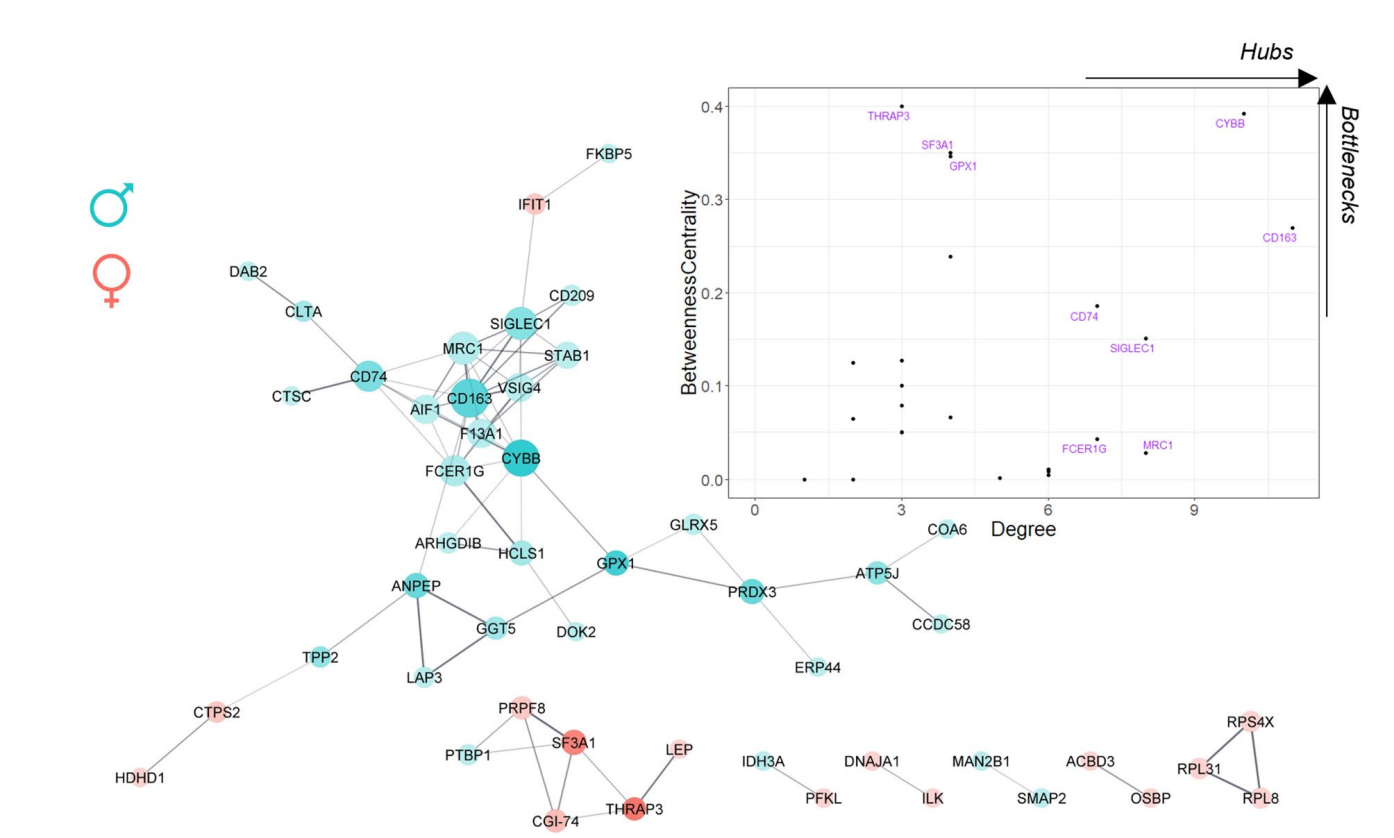
Protein-protein interaction analysis of all 74 dysregulated proteins. The network was built with Cytoscape, sourcing the STRING database (v11.5). Only interactions with a confidence score greater than 0.4 were considered. Thicker edges represent more confident interactions. Proteins upregulated in men and women are depicted by turquoise and salmon nodes, respectively. Proteins are identified by the UniProt-recommended gene name. Using topological features, nodes were styled to reflect the degree and centrality: size is proportional to the degree, and opacity is proportional to betweenness centrality. The plot in the top right corner distributes the proteins according to their topological properties. Proteins with a more hub and/or bottleneck profile are labelled

STRING analysis was carried out to dissect how proteins were functionally grouped and to determine hubs (high node degree) and bottleneck (high betweenness centrality) proteins, which may be master regulators of sex-specific pathways that drive AVS (Fig. 4). Topological network analysis identified a major cluster essentially comprising proteins upregulated in men, mostly leukocyte-associated. CD163, a well-known monocyte/macrophage marker, was the central-most protein. Other leukocyte markers that were increased in men were CD74, a marker of macrophages, dendritic cells, and B cells, and CD209, a marker of dendritic cells. NADPH oxidase 2 (CYBB/NOX2) also occupied a central position in the network. NOX2 generates superoxide in phagocytes, being a major contributor to oxidative stress. Other immune system cell-related proteins identified in the major cluster included glutathione peroxidase 1 (GPX1), a key antioxidant defence enzyme, and the aminopeptidase N (ANPEP), a multifunctional peptidase with active roles in antigen presentation, peptide hormone processing and potentially in cholesterol crystallisation. Two smaller clusters (with six and three elements) were evident, mainly composed of proteins upregulated in the women’s valves. In both cases, these were ribosomal proteins such as 60 S ribosomal protein L31 (RPL31) and 8 (RPL8) or spliceosome proteins, such as splicing factor 3 A subunit 1 (SF3A1), in line with the enrichment analysis of cellular components.

An exploratory correlation analysis of the proteome with disease severity is given in the Supplementary Material (Supplementary Fig. 4 and Supplementary Files 3 and 4).

### Validation of the sexual dimorphism at the transcript level

**Fig. 5:**
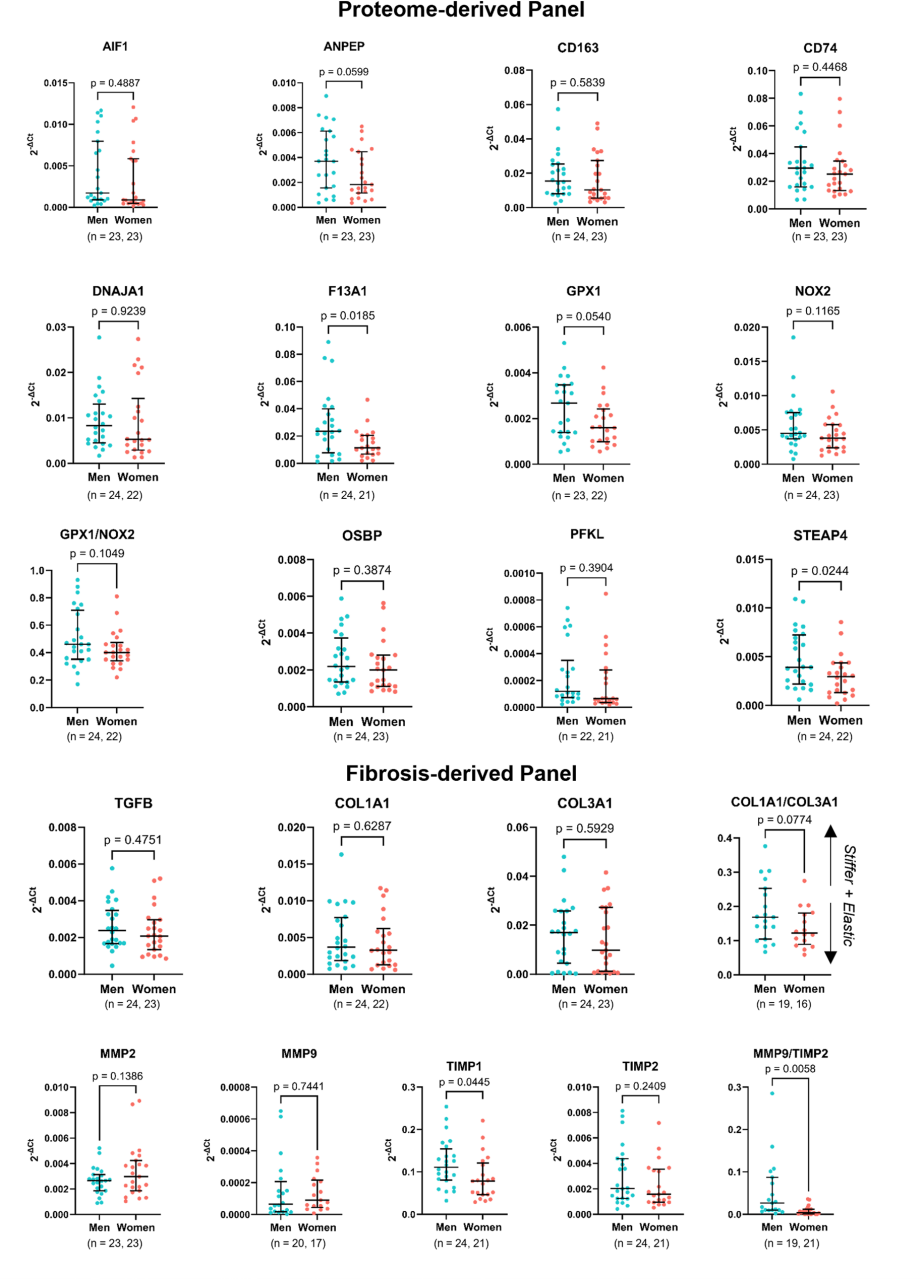
Validation of specific targets at the transcript level by qRT-PCR. The quantification of the gene expression of a selected proteome-derived panel (AIF1, ANPEP, CD163, CD74, DNAJA1, F13A1, GPX1, NOX2, OSBP, PFKL, STEAP4) and of a fibrosis-related panel (TGFB, COL1A1, COL3A1, MMP2, MMP9, TIMP1, TIMP2) is shown, respectively, on top and bottom. Gene expression is normalised to RNA18S. The GPX1/NOX2, COL1A1/COL3A1 and MMP9/TIMP2 ratios are also presented. In all cases, data are presented as median and interquartile range and individual biological replicates as dots (n is indicated for each transcript). Differences were inquired with an unpaired t-test

Some DEPs were selected for subsequent validation (Supplementary Table 6). After repeated attempts, we found several proteins below or near the detection limit of western blotting, which would result in unreliable quantification. Thus, sex differences were validated at the mRNA level (Fig. 5, top) and by other protein-targeted assays (following sections). We confirmed that the coagulation factor XIII A chain (*F13A1*) and metalloreductase *STEAP4* were already significantly increased at the transcript level in men’s valves. An apparent trend for higher transcript levels of *ANPEP* (*p* = 0.06) and *GPX1* (*p* = 0.05) was also observed in men. Regarding the remaining analytes, the differences may be limited to the protein level, as the transcripts did not differ between sexes. Moreover, we found sex-specific correlations between gene expression levels and disease severity parameters, further confirming sexual dimorphism (Supplementary Table 7).

Given the staggering difference in valve fibrosis, we also assessed the levels of a fibrosis-related gene panel (Fig. 5, bottom). No differences were observed in *TGFB*, a master regulator of fibrosis, or genes coding for collagens, *COL1A1*, or *COL3A1*. However, a higher *COL1A1*/*COL3A1* ratio was found in men, although not reaching statistical significance (*p* = 0.08). *MMP2* and *MMP9*, major players in extracellular matrix (ECM) degradation, were not differentially expressed between sexes. In turn, women presented a significantly lower expression of tissue inhibitor of metalloproteinases 1 (*TIMP1*) but not *TIMP2*. A significantly lower *MMP9*/*TIMP2* ratio was also observed in women.

### CD74 immunohistochemistry

**Fig. 6:**
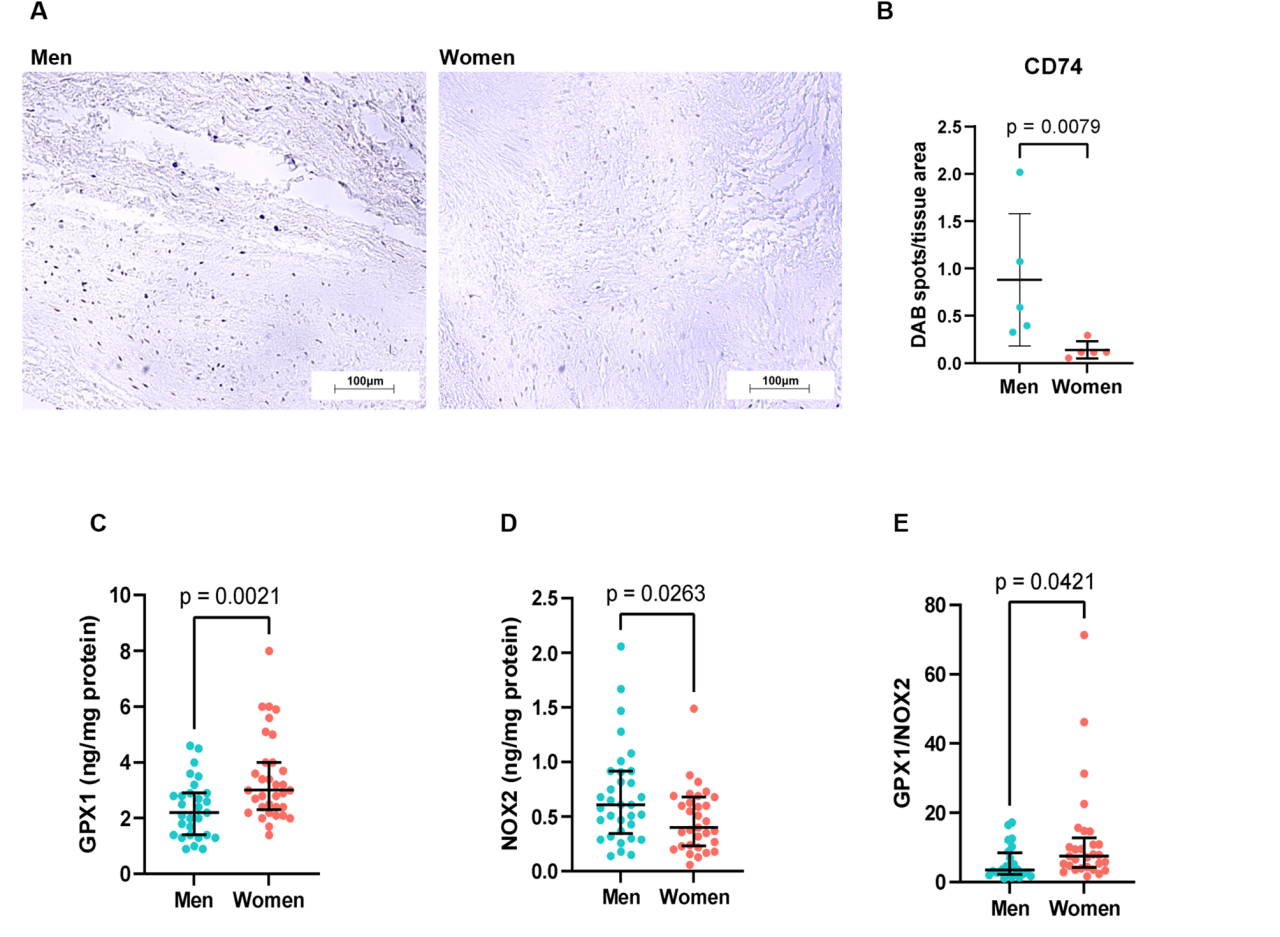
Validation of the differential expression of specific proteins by immunohistochemistry and ELISA. (**A**) Representative aortic valve cross-sections (20x magnification) from a man and woman with aortic valve stenosis immunostained against CD74. (**B**) Quantification of CD74 expressed as % of DAB spots to total tissue area (*n* = 5 men, *n* = 5 women). Differences were inquired with a Mann-Whitney test. (**C**) Quantification of GPX1 (*n* = 29 men, *n* = 33 women) and (**D**) NOX2 in aortic valve tissue lysates (*n* = 33 men, *n* = 31 women) by ELISA, normalised to the total protein amount. (**E**) Analysis of the antioxidant/pro-oxidant balance through the GPX1/NOX2 ratio (*n* = 29 men, *n* = 24 women). In all cases, data are presented as median and interquartile range and individual biological replicates as dots. From C to E, differences were inquired with an unpaired t-test

Through proteomics, we found differential expression of three leukocyte markers between the sexes, specifically CD74, CD163, and CD209. In any case, these levels were increased in the valves of men, suggesting amplified leukocyte infiltration. CD74 and CD163 were the most central proteins in the PPI network and were thus deemed functionally the most relevant. Therefore, we aimed to validate the differences in these proteins using immunohistochemistry. CD163 could not be successfully assessed, but CD74 expression was more pronounced in men (Fig. 6A-B), corroborating the increased infiltration of macrophages, dendritic cells, and B cells into their valves.

### GPX1 and NOX2 quantification by ELISA

GPX1 and NOX2 are among other proteins upregulated in men’s valves through proteomics but deserve special attention. Indeed, GPX1 and NOX2 occupy a central position in the PPI network, suggesting a pivotal role in the definition of sex differences in AVS. They have opposing effects on oxidative stress, with GPX1 exerting antioxidant effects and NOX2 acting as a pro-oxidant. Hence, we decided to quantify these proteins further using ELISA in an extended cohort of 80 patients (Fig. 6C-E). In contrast to the proteomic data, GPX1 levels were significantly increased in the women’s valves (1.5-fold, *p* < 0.01). In turn, NOX2 was found to be increased in men’s valves (1.5-fold, *p* < 0.05), in accordance with proteome analysis. Importantly, when considered together, a significantly higher GPX1/NOX2 ratio (2.2-fold, *p* < 0.05) was found in women, suggesting a higher shield against oxidative stress.

In addition, GPX1 was found to be inversely correlated with the taoGmean in men (*r*=−0.50, *p* < 0.01) but positively correlated with LV hypertrophy (*r* = 0.57, *p* < 0.01). For NOX2, no correlations were observed.

## Discussion

There is a clear clinical need for improvement in the management and treatment of AVS. Despite the efforts of several clinical trials (some still ongoing) targeting, e.g., PCSK9, lipoprotein(a), or matrix-Gla protein, no pharmacotherapy has yet been proven to reverse/halt AVS progression [6]. Currently, the only option for the diagnosed patients is to wait until they are eligible for AVR, which entails the risk of complications, irreversible myocardial damage, and high procedural costs [2]. While our knowledge of the mechanisms of the disease is mounting with the help of different omic approaches, there is still a significant gap regarding sexual dimorphism. Therefore, we characterised, for the first time, sexual dimorphism in AVS at the proteome level (Graphical Abstract) to lay the foundation for the future development of sex-specific therapies for AVS treatment.

First, we confirmed the sexual dimorphism at the histological level. As expected, women presented more significant valve fibrosis, and men demonstrated greater calcification, as previously reported [14, 29]. We also confirmed that valves excised from men were heavier after adjusting for body size or AVA. Valve weight and the degree of calcification assessed by CT are strongly correlated [30]. Therefore, although we did not score calcium, gross analysis and histochemistry confirmed that men’s valves were more calcified despite similar clinical stenosis severity (Table 1). Previously, Simard et al. [14]. showed that for the same haemodynamic severity, men present with higher valve calcification and women present with higher valve fibrosis.

The high extent of collagenous tissue in women cannot be explained by the higher expression of *TGFB*, the master regulator of fibrosis. Nonetheless, a trend for a lower *COL1A1*/*COL3A1* ratio was found in women. This aligns with a previous study showing that women have a similar protein expression of COL1A1 and a higher expression of COL3A1 [31]. This imbalance towards collagen I in men might additionally contribute, beyond calcification, to a higher valve stiffness. We also found a significantly higher expression of *TIMP1* mRNA in men’s valves. In agreement, male porcine VICs were previously reported to express significantly higher levels of *TIMP1* mRNA despite the lower protein levels [32]. We also observed a lower *MMP9*/*TIMP2* ratio in women, promoting net preservation and ECM build-up. Intriguingly, we did not observe higher *MMP9* gene expression in men. This might be explained by protein-level differences or differences exclusive to the inflammatory leukocyte infiltrate, which is likely obscured by whole-tissue analysis.

Bioinformatics analysis further suggests differences in valvular cell composition between sexes in AVS. Although no specific cell types were particularly prominent in women’s valves, the men’s valves were enriched in proteins indicative of the presence of cells of myeloid origin, including monocytes, macrophages and dendritic cells. This proteomic signature confirms the transcriptomic signature reported by Myasoedova et al. [8]., who found that men’s valves were enriched with immune cells (monocytes, macrophages, dendritic cells, B and T cells), whereas women’s are enriched with chondrocytes, fibroblasts, osteoblasts, and pericytes. The lack of women-specific cell types in our analysis is explained by the larger effect of the proteins directly involved in immune system activity, which passed our filtering strategy (Methods). Indeed, when we rerun the enrichment analysis with all proteins increased in women (an exploratory analysis, without *p*-value adjustment), a significant enrichment of smooth muscle cells was found (Supplementary Table 8), which suggests the presence of myofibroblasts (smooth muscle-like fibroblasts) resulting from VICs activation [33].

Such a sex-asymmetric distribution of cell types was also observed in FEA. Previously, pathways overactivated in women, remarkably mRNA splicing regulation, accounted for 25% of all dysregulated pathways by transcriptomics [8]. Our data corroborate these findings, showing that proteins localised in the spliceosomal complex and the ribosome are particularly increased in women. This also explains why not all dysregulated proteins were confirmed at the transcript level. Alternative splicing of transcripts in women may explain, at least in part, sexual dimorphism and merits further scrutiny. Beyond the spliceosome, we found even more significant dysregulation of proteins localised in focal adhesions, critical mechano-sensing structures. Previously, the regulation of the Rho/Rho-associated protein kinase signalling pathway, involved in the transduction of ECM mechanical cues and in the differentiation of VICs to myofibroblasts, was reported to be different in women’s VICs [34]. These differences underscore the relevance of tailoring new molecular therapies to each sex. Our study points to focal adhesions as an interesting hotspot for mitigating AVS disease in women.

As for cells, the bulk of enriched gene ontology terms translated into men-specific dysregulation. Overall, FEA demonstrates a marked immune system activation in men’s valves, emphasising monocyte activation into macrophages and subsequent antigen processing and presentation of peptides to initiate adaptive immunity. Indeed, innate and adaptive immune responses were the largest cluster associated with men by transcriptomics [8]. We confirmed by immunohistochemistry a higher CD74 expression in men’s valves. CD74 plays a crucial role in major histocompatibility complex class II antigen processing and is a marker of antigen-presenting (macrophages and dendritic cells) and B cells. CD74 occupied a central position in the PPI network, corroborating a previous bioinformatics analysis that reported CD74 among the top 10 hub genes in a PPI analysis of AVS transcriptome data [35]. CD74 is also relevant in other atherosclerotic conditions, such as carotid stenosis, being correlated with intima-media thickness [36]. The valve proteome also showed an increased CD163 expression, which was not observed at the transcript level. Unfortunately, we could not confirm this by immunohistochemistry, probably because of shedding and subsequent release in a soluble form (sCD163).

FEA also showed an enrichment of proteins involved in lipoprotein binding, specifically LDL, and scavenger receptor activity in men, suggesting that the engulfment of accumulated lipoproteins and other products by macrophages was more pronounced in these patients. In addition, we found with proteomics and PCR confirmed that men’s valves have a higher expression of F13A1. The latest evidence points to a pathogenic role for F13A in transforming lipid-laden macrophages into foam cells. Somodi et al. [37]. reported a macrophage-specific accumulation of F13A1 in carotid plaques, concomitant with increased protein cross-linking activity, which might have a role in plaque stabilisation. Moreover, we found increased ANPEP expression at the transcript and protein levels in men’s valves. ANPEP has previously shown a concentration-dependent cholesterol crystallisation activity in vitro [38]. Hence, dissecting whether targeted inhibition of this aminopeptidase and/or of F13A1 in the aortic valve would stall AVS progression in men would be interesting.

The disruption of the aortic valve endothelial layer is widely accepted to be on the inception of AVS [6]. Endothelial micro-fissures promote the infiltration of lipoproteins and circulating cells, such as red blood cells (RBCs) [39]. Upon exposure to RBC degradation products, such as haemoglobin and the free oxidised iron (Fe^3+^), VICs acquire a pro-osteogenic phenotype, leading to reduced proliferation, decreased elastin production and enhanced calcium deposition [40, 41]. CD163, also known as the haemoglobin scavenger receptor, is responsible for clearing haemoglobin to prevent oxidative damage. CD163 and haemoglobin cohabitate strongly in calcified areas [42]. In our analysis, CD163 occupied a highly central position in a PPI network (highest node degree). We observed increased CD163 expression in men (proteomics), suggesting that men’s valves are more prone to RBC accumulation. Higher enrichment of endocytosis-related proteins in men also suggests increased phagocytosis to clear lipoproteins and RBC. Moreover, we observed an increase of the metalloreductase STEAP4 at the transcript and protein level in men’s valves. STEAP4 is an NADPH-dependent enzyme that reduces Fe^3+^ to Fe^2+^; therefore, it may play a key role in protecting against RBC lysis-induced iron release and subsequent oxidative stress. This effect needs to be confirmed in the future, as we cannot establish causality; however, STEAP4 has been shown to exert an anti-inflammatory and atheroprotective role, regulating NADPH levels in macrophages and cell foam formation [43].

Our proteomic approach also identified oxidative stress as a major difference between sexes in AVS. NOX2, which generates superoxide anion, was found to be increased in men by proteomics and by ELISA in a larger cohort. GPX1, which is responsible for hydrogen peroxide neutralisation, was found to be increased in men by proteomics but not by ELISA in an extended cohort. Instead, we observed an increase in GPX1 levels and a higher GPX1/NOX2 ratio in women’s valve lysates, suggesting lower levels of oxidative stress in women. Recently, a lower expression of oxidative stress markers, such as endothelial nitric oxide synthase, myeloperoxidase, malondialdehyde and nitrotyrosine, was reported in stenotic valves from women [31]. Considering that GPX1 ELISA quantification was absolute and based on dual antibody recognition, we believe GPX1 mass spectrometry semi-quantification was inaccurate. The trend for increased GPX1 transcripts in men’s valves might be explained by a chronic response to overt oxidative stress, with exhaustion of the protein antioxidant agents. Targeting oxidative stress and, thus, calcification could be a more effective strategy for treating AVS in men. In fact, it has been previously demonstrated that celastrol, a NOX2 inhibitor, could reduce ROS generation and calcium deposition, sided with an improvement of left ventricular dilatation in a calcific aortic valve disease rabbit model [44].

### Study limitations

The present study was exploratory. No power analysis was performed, but only proteins with a large effect size were considered DEPs. The main limitation is the lack of a disease-free control group, which prevents us from assigning sex-dysregulated proteins to healthy or diseased states. However, all associated pathways (fibrosis/ECM remodelling, inflammation and immune system activation, phagocytosis, and oxidative stress) have been previously reported in AVS progression. Whole-tissue analysis also hinders the confirmation of cell-specific targets for a more directed sex-specific therapy. Cell type enrichment analysis requires further validation by flow cytometry or other cell-specific assays. Nonetheless, our proteomic analysis provides some clues regarding specific immune cell clusters that may be preferred targets in men (e.g., CD74 and CD163). Men and women were not matched for the presence of bicuspid valves, but the incidence was similar between sexes across the cohorts. Finally, even though virtually all women included were postmenopausal, we did not evaluate patients’ hormone status, and this can modulate the aortic valve phenotype [45].

## Conclusions

AVS is characterised by a clear sex dimorphism at the histological and proteomic levels. Among patients with severe stenosis, women display more extensive ECM remodelling and are more prone to fibrosis, with subsequent dysregulation of proteins localised in focal adhesions. Alternative splicing of transcripts can explain women-specific aortic valve remodelling patterns. In turn, men show a higher propensity for calcification, which might be explained by their proclivity for the accumulation of circulating red and white blood cells, lipoproteins, and other molecules that build up and oxidise, accelerating the osteogenic transformation of VICs. Using proteomics, we systematically uncovered the pathways associated with sex dimorphism in AVS and unveiled protein players emerging as surrogate therapeutic targets for a sex-personalised modulation of the disease.

## Electronic supplementary material

Supplementary Material 1.

Supplementary Material 2.

Supplementary Material 3.

Supplementary Material 4

Supplementary Material 5

## Data Availability

The dataset supporting the conclusions of this article is available in the PRIDE repository, with the identifier PXD051201.

## Abbreviations

AVA: aortic valve area
AVR: aortic valve replacement
AVS: aortic valve stenosis
CT: computed tomography
DAB: 3,3’-diaminobenzidine
DEPs: differentially expressed proteins
DIA: data-independent acquisition
DVI: Doppler velocity index
ECM: extracellular matrix
FEA: functional enrichment analysis
H&E: haematoxylin and eosin
LV: left ventricle
MS: mass spectrometry
PPI: protein-protein interaction
RBCs: red blood cells
taoGeman: mean transvalvular aortic pressure gradient
taoGmax: maximal transvalvular aortic pressure gradient
VICs: valve interstitial cells
