# Supplementary material for: Dissecting sexual dimorphism in aortic valve stenosis by proteomics
Source: Clin Proteomics. 2025 Oct 6;22:34. doi: 10.1186/s12014-025-09549-1 (PMC12502187; doi:10.1186/s12014-025-09549-1)
Supplement: Supplementary file 5 — Supplementary Material 5 [file 12014_2025_9549_MOESM5_ESM.docx]

Dissecting Sexual Dimorphism in Aortic Valve Stenosis by Proteomics

Ana Grego^1^, Cláudia Sousa-Mendes^1^, Diana Martins^1^, Carla Sousa^1,2^, Ana Filipa Ferreira^1^, Francisca Saraiva^1^, Inês Alves^1^, Guadalupe Espadas^3,4^, Isabel Miranda^1^, Adelino Leite-Moreira^1,5^, Eduard Sabidó^3,4^, António S. Barros^1^, Cristina Gavina^1,6^, Rui Vitorino^1,7,8^, Inês Falcão-Pires^1^, Rita Nogueira-Ferreira^1^, Fábio Trindade^1*^

^1^ Cardiovascular R&D Centre - UnIC@RISE, Department of Surgery and Physiology, Faculty of Medicine of the University of Porto, 4200-319 Porto, Portugal

^2^ Cardiology Department, University Hospital Center São João, 4200-319 Porto, Portugal

^3^ Proteomics Unit, Center for Genomic Regulation, Barcelona Institute of Science and Technology (BIST), 08003, Barcelona, Spain

^4^ Proteomics Unit, Universitat Pompeu Fabra, 08003, Barcelona, Spain

^5^ Department of Cardiothoracic Surgery, University Hospital Center São João, 4200-319 Porto, Portugal

^6^ Cardiology Department, Pedro Hispano Hospital, 4464-513 Matosinhos, Portugal

^7^ iBiMED – Institute of Biomedicine, Department of Medical Sciences, University of Aveiro, 3810-193 Aveiro, Portugal

^8^ LAQV/REQUIMTE, Department of Chemistry, University of Aveiro, 3810-193 Aveiro, Portugal

*Corresponding author

Fábio Trindade

[ftrindade@med.up.pt](mailto:ftrindade@med.up.pt)

Cardiovascular R&D Centre - UnIC@RISE

Department of Surgery and Physiology, Faculty of Medicine of the University of Porto

4200-319 Porto, Portugal

**Supplementary Figures and Analyses**


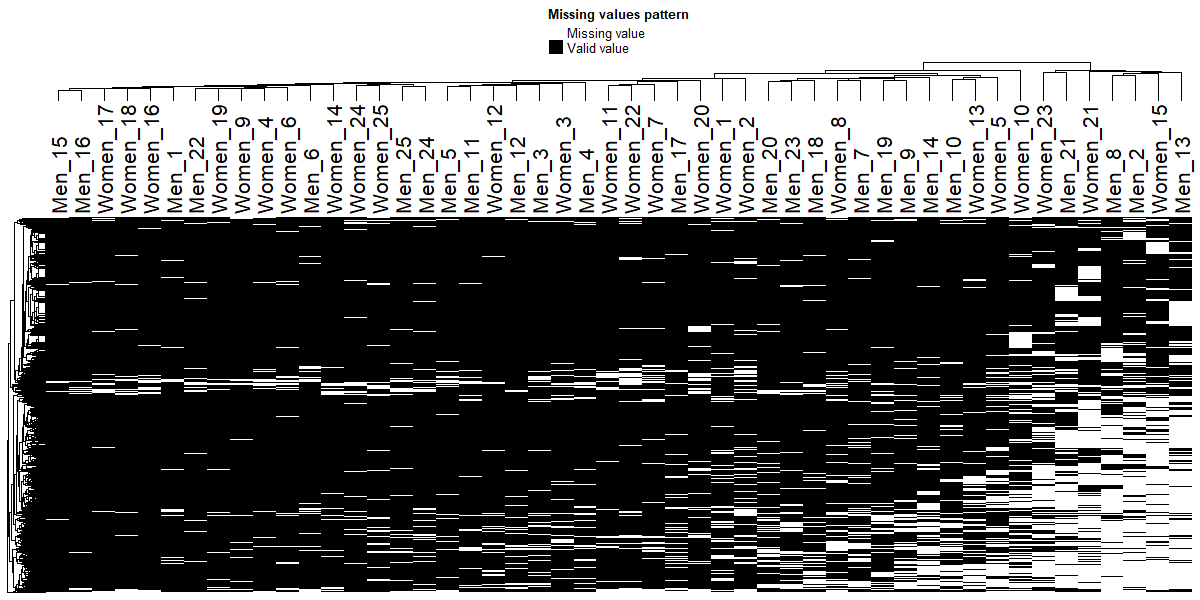


**Fig. S1.** Missing data analysis. Heatmap showing the clustering of samples according to the protein quantification missing values (white). No sex-specific clusters were formed. Thus, missing values were not biased to any sex.


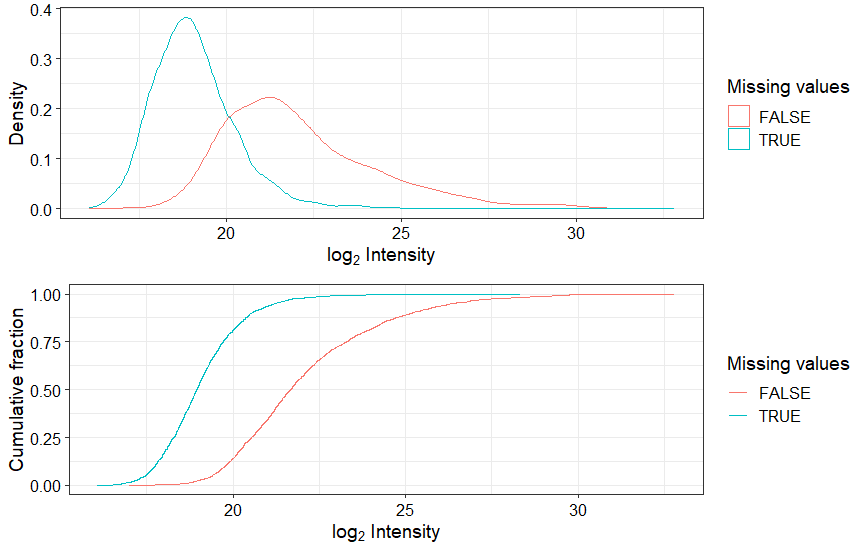


**Fig. S2.** Missing data analysis. Density and cumulative fraction plots of the protein quantification data, showing that missing values corresponded to proteins closer to the detection limit (log2 intensity <20), as expected. We did not impute these values because a robust acquisition method was used (data-independent acquisition).

**Fig. S3.** Comparison of the excised valve weight, normalised to aortic valve area (AVA), between men (n = 24) and women (n = 23) undergoing surgical valve replacement.

**Correlation with disease severity**

Sex differences in disease presentation were further evidenced by the correlations between protein levels and disease severity. Very few proteins common to both sexes were found to correlate with the AVA, mean transvalvular aortic pressure gradient (taoGmean), DVI or LV mass (**Supplementary Fig. 4A**), and none were among the top 10 positively and the top 10 inversely correlated with disease severity (**Supplementary** **Figs. 4B-4I**). Notably, the U5 small nuclear ribonucleoprotein 40 kDa protein (SNRNP40) and the microsomal glutathione S-transferase 2 (MGST2) were found to correlate simultaneously with the three main parameters of disease severity, AVA, taoGmean and DVI, in men. In women, SLC35A4 (probable UDP-sugar transporter), stromal cell-derived factor 2-like protein 1 (SDF2L1), methylcrotonoyl-CoA carboxylase beta chain, mitochondrial (MCCC2), ATP synthase F(0) complex subunit B1, mitochondrial (ATP5PB), inter-alpha-trypsin inhibitor heavy chain H5 (ITIH5) and latent-transforming growth factor beta-binding protein 2 (LTBP2) were simultaneously associated with the same three parameters (**Supplementary Files 3** and **4** compile all correlations for men and women). The almost complete lack of overlapping correlations further demonstrates the activation of divergent pathways in men and women during the course of the disease.


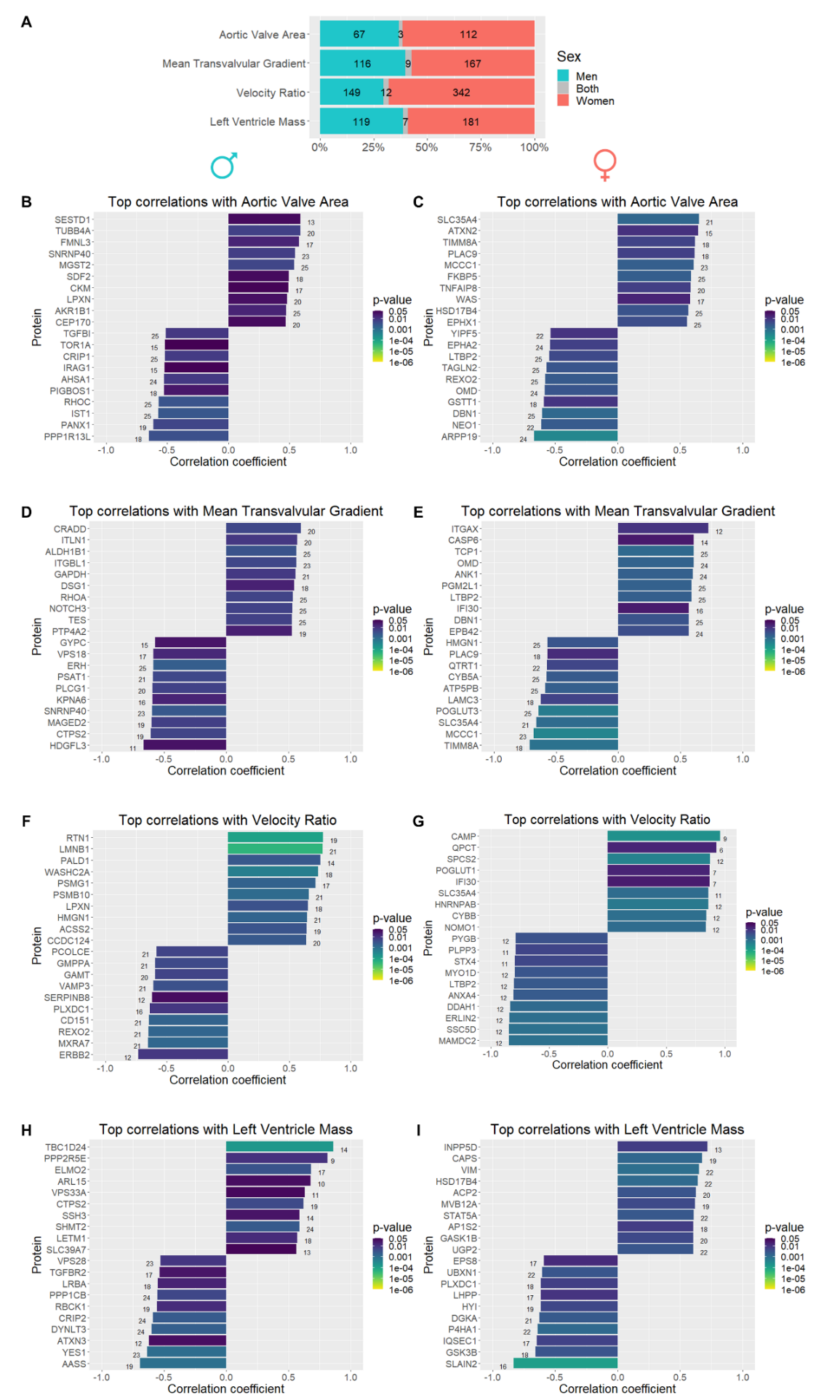


**Fig. S4.** Correlations between aortic valve proteome and disease severity. A) Sum of all proteins correlating with the most relevant disease severity parameters, showing that only a minor fraction of the associations is sex-unspecific. B) to I) Top 20 proteins (10 directly and 10 inversely) correlating with aortic valve area [B), men; C), women], mean transvalvular pressure gradient [D), men; E), women], velocity ratio [F), men; G), women] and left ventricle mass [H), men; I), women]. The numbers beside the bars indicate the number of data pairs. In all cases, Spearman test was used. The bars’ colour represents the significance of the correlation. All proteins quantified (3,980) were included in this analysis.

**Supplementary Tables**

**Supplementary Table 1.** Sequences of the primer pairs used for PCR.

| **Gene name** | **Direction** | **Primer name** | **Sequence (5’ – 3’)** |
| --- | --- | --- | --- |
| AIF1 | Forward | HsAIF1_F | CCAGGGATTTACAGGGAGGA |
|  | Reverse | HsAIF1_R | GGAGGGCAGATCCTCATCAC |
| ANPEP | Forward | HsANPEP_F | GGGCAATGTCAGAAAGGTGG |
|  | Reverse | HsANPEP_R | GGTCCTTGGGGTGGATAAGC |
| CD163 | Forward | HsCD163_F | TAACCCAGTGAGTTCAGCCT |
|  | Reverse | HsCD163_R | ATTCAGCAGCAGTCTTAGGAA |
| CD74 | Forward | HsCD74_F | CTGCTGGTGTGTCTTCCCC |
|  | Reverse | HsCD74_R | GCTCTCACATGGGGACTGG |
| DNAJA1 | Forward | HsDNAJA1_F | ACCCAAATGAAGGAGAGAAGTTTA |
|  | Reverse | HsDNAJA1_R | CACCTGCTCCACCCTCTTTA |
| F13A1 | Forward | HsF13A1_F | GTGGGGTCTGCAATGGTGAA |
|  | Reverse | HsF13A1_R | ATGTCAACGCTTCCAGTCCA |
| GPX1 | Forward | HsGPX1_F | CCGGGACTACACCCAGATGA |
|  | Reverse | HsGPX1_R | TCTTGGCGTTCTCCTGATGC |
| MMP2 | Forward | HsMMP2_F | CTCCCCATGAAGCCCTGTTC |
|  | Reverse | HsMMP2_R | GCTGTCATAGGATGTGCCCT |
| MMP9 | Forward | HsMMP9_F | GGCGCTCATGTACCCTATGT |
|  | Reverse | HsMMP9_R | TTCAGGGCGAGGACCATAGA |
| NOX2 | Forward | HsNOX2_F | AACTGGGCTGTGAATGAGGG |
|  | Reverse | HsNOX2_R | GCCAGTGCTGACCCAAGAA |
| OSBP1 | Forward | HsOSBP1_F | CTCCTGGCAATGTGGGTTCT |
|  | Reverse | HsOSBP1_R | TTGCTGCCAGTACGTTTGTG |
| PFKL | Forward | HsPFKL_F | GACGCGCAAGGCATGA |
|  | Reverse | HsPFKL_R | CCAGTTGGCCTGCTTGATGT |
| STEAP4 | Forward | HsSTEAP4_F | TTGCTCATTTGGTGCCAGGA |
|  | Reverse | HsSTEAP4_R | TCCACACACAAACACCTGCC |
| COL1A1 | Forward | HsCOL1A1_F | CTCTGGTCCTCGTGGTCT |
|  | Reverse | HsCOL1A1_R | CCCCATCATCTCCATTCTTTCC |
| COL3A1 | Forward | HsCOL3A1_F | GGTGCTAATGGTGCTCCT |
|  | Reverse | HsCOL3A1_R | TCCTTGCCATCTTCGCCTTT |
| TGFB | Forward | HsTGFB_F | GAAGAACTGCTGCGTGCG |
|  | Reverse | HsTGFB_R | GTGTCCAGGCTCCAAATGT |
| TIMP1 | Forward | HsTIMP1_F | CTGTTGGCTGTGAGGAATGC |
|  | Reverse | HsTIMP1_R | AGGTGACGGGACTGGAAG |
| TIMP2 | Forward | HsTIMP2_F | GAGCACCACCCAGAAGAAG |
|  | Reverse | HsTIMP2_R | CAGTCCATCCAGAGGCAC |
| RNA18SN1 | Forward | Hs18S_F | GACTCAACACGGGAAACCTC |
|  | Reverse | Hs18S_R | CCAGACAAATCGCTCCACC |

**Supplementary Table 2.** Characteristics of the validation populations.

|  | **qRT-PCR cohort** | | | | **Immunohistochemistry cohort** | | | | **ELISA cohort** | | | |  |
| --- | --- | --- | --- | --- | --- | --- | --- | --- | --- | --- | --- | --- | --- |
|  | **N** | **men**,  N = 24^a^ | **women**,  N = 23^a^ | **p-value**^b^ | **N** | **men**,  N = 6^1^ | **women**,  N = 6^a^ | **p-value^c^** | **N** | **men**,  N = 40^a^ | **women**,  N = 40^a^ | **p-value**^b^ |  |
| Age at AVR | 47 | 73 (69, 76) | 71 (65, 77) | 0.7 | 12 | 69 (63, 77) | 70 (64, 75) | 0.7 | 80 | 72 (66, 76) | 74 (67, 77) | 0.5 |  |
| BMI (kg/m^2^) | 47 | 27.6 (26.6, 29.0) | 31.0 (27.9, 32.7) | **0.003** | 12 | 28.83 (27.93, 29.28) | 30.73 (28.25, 32.93) | 0.2 | 80 | 27.1 (25.8, 29.1) | 29.1 (27.0, 32.2) | **0.020** |  |
| ***Comorbidities*** |  |  |  |  |  |  |  |  |  |  |  |  |  |
| Dyslipidaemia | 47 | 20 (83%) | 15 (65%) | 0.2 | 12 | 4 (67%) | 4 (67%) | >0.9 | 80 | 30 (75%) | 29 (73%) | 0.8 |  |
| Smoking habits | 47 | 6 (25%) | 0 (0%) | **0.022** | 12 | 3 (50%) | 0 (0%) | 0.2 | 80 | 12 (30%) | 2 (5.0%) | **0.003** |  |
| Arterial hypertension | 47 | 20 (83%) | 21 (91%) | 0.7 | 12 | 6 (100%) | 6 (100%) | >0.9 | 80 | 32 (80%) | 33 (83%) | 0.8 |  |
| Diabetes mellitus | 47 | 12 (50%) | 7 (30%) | 0.2 | 12 | 5 (83%) | 1 (17%) | 0.080 | 80 | 17 (43%) | 13 (33%) | 0.4 |  |
| CAD | 47 | 7 (29%) | 5 (22%) | 0.6 | 12 | 1 (17%) | 0 (0%) | >0.9 | 80 | 11 (28%) | 7 (18%) | 0.3 |  |
| AR (mild-moderate) | 47 | 16 (67%) | 16 (70%) | 0.8 | 12 | 4 (67%) | 5 (83%) | >0.9 | 80 | 26 (65%) | 26 (65%) | >0.9 |  |
| ***Pharmacology*** |  |  |  |  |  |  |  |  |  |  |  |  |  |
| Statins | 47 | 23 (96%) | 14 (61%) | **0.004** | 12 | 6 (100%) | 4 (67%) | 0.5 | 79 | 31 (79%) | 28 (70%) | 0.3 |  |
| Beta-blockers | 47 | 11 (46%) | 11 (48%) | 0.9 | 12 | 3 (50%) | 3 (50%) | >0.9 | 79 | 15 (38%) | 15 (38%) | >0.9 |  |
| ACEi | 47 | 6 (25%) | 9 (39%) | 0.3 | 12 | 2 (33%) | 3 (50%) | >0.9 | 79 | 9 (23%) | 15 (38%) | 0.2 |  |
| AT2Ri | 47 | 12 (50%) | 10 (43%) | 0.7 | 12 | 3 (50%) | 2 (33%) | >0.9 | 79 | 18 (46%) | 16 (40%) | 0.6 |  |
| Diuretics | 47 | 11 (46%) | 16 (70%) | 0.10 | 12 | 3 (50%) | 6 (100%) | 0.2 | 79 | 19 (49%) | 23 (58%) | 0.4 |  |
| Anticoagulants | 47 | 2 (8.3%) | 2 (8.7%) | >0.9 | 12 | 0 (0%) | 1 (17%) | >0.9 | 79 | 3 (7.7%) | 2 (5.0%) | 0.7 |  |
| Aspirin | 47 | 13 (54%) | 6 (26%) | **0.050** | 12 | 4 (67%) | 2 (33%) | 0.6 | 79 | 21 (54%) | 14 (35%) | 0.092 |  |
| CAC blockers | 47 | 9 (38%) | 10 (43%) | 0.7 | 12 | 3 (50%) | 1 (17%) | 0.5 | 79 | 15 (38%) | 15 (38%) | >0.9 |  |
| SGLT2i | 47 | 18 (75%) | 22 (96%) | 0.10 | 12 | 3 (50%) | 6 (100%) | 0.2 | 79 | 33 (85%) | 39 (98%) | 0.057 |  |
| ***Disease severity*** |  |  |  |  |  |  |  |  |  |  |  |  |  |
| taoGmax (mmHg) | 45 | 74 (62, 85) | 81 (77, 91) | 0.11 | 12 | 86 (78, 98) | 80 (79, 80) | 0.7 | 78 | 73 (63, 83) | 80 (69, 90) | 0.10 |  |
| taoGmean (mmHg) | 47 | 45 (40, 55) | 51 (44, 60) | 0.2 | 12 | 59 (47, 68) | 51 (49, 53) | 0.4 | 80 | 45 (40, 52) | 50 (43, 59) | 0.13 |  |
| AVAi (cm^2^/m^2^) | 47 | 0.46 (0.42, 0.52) | 0.43 (0.37, 0.48) | 0.2 | 12 | 0.42 (0.37, 0.46) | 0.44 (0.41, 0.49) | 0.6 | 80 | 0.47 (0.43, 0.53) | 0.46 (0.39, 0.50) | 0.2 |  |
| DVI | 33 | 0.22 (0.18, 0.25) | 0.25 (0.22, 0.26) | 0.079 | 7 | 0.22 (0.20, 0.25) | 0.25 (0.25, 0.26) | 0.4 | 54 | 0.22 (0.20, 0.25) | 0.24 (0.21, 0.25) | 0.3 |  |
| LVMi (g/m^2^) | 42 | 115 (104, 142) | 115 (103, 135) | 0.9 | 10 | 132 (110, 140) | 134 (113, 145) | 0.5 | 74 | 121 (108, 143) | 118 (104, 140) | 0.6 |  |
| Bicuspid valves | 46 | 6 (25%) | 7 (30%) | 0.8 | 12 | 2 (33%) | 2 (33%) | >0.9 | 75 | 12 (31%) | 13 (36%) | 0.6 |  |
| ^a^n (%); Median (IQR); ^b^Wilcoxon rank sum test; Pearson's Chi-squared test; Fisher's exact test; ^c^Wilcoxon rank sum test; Wilcoxon rank sum exact test; Fisher's exact test | | | | | | | | | | | | | |
| ACEi – angiotensin-converting enzyme inhibitors; AR – aortic regurgitation; AT2Ri – angiotensin II receptor inhibitors; AVAi – aortic valve area indexed to body surface area; AVR – aortic valve replacement; BMI – body mass index; CAC blockers – calcium channel blockers; CAD – coronary artery disease; DVI – Doppler velocity index (velocity ratio); LVMi – left ventricle mass indexed to body surface area; SGLT2i – sodium-glucose co-transporter 2 inhibitors; taoGmax – maximal transvalvular aortic pressure gradient; taoGmean – mean transvalvular aortic pressure gradient | | | | | | | | | | | | | |

**Supplementary Table 3**. Characteristics of the ELISA cohort (patients whose protein could effectively be quantified).

|  | **GPX1 ELISA cohort** | | | | **NOX2 ELISA cohort** | | | |
| --- | --- | --- | --- | --- | --- | --- | --- | --- |
|  | **N** | **men**, N = 29^a^ | **women**, N = 33^a^ | **p-value**^b^ | **N** | **men**, N = 34^a^ | **women**, N = 34^a^ | **p-value**^b^ |
| Age at AVR | 62 | 71 (65, 75) | 75 (68, 77) | 0.077 | 68 | 71 (66, 76) | 73 (67, 77) | 0.4 |
| BMI (kg/m^2^) | 62 | 27.1 (26.3, 29.1) | 28.7 (27.1, 31.6) | 0.07 | 68 | 27.3 (25.6, 29.4) | 29.2 (26.1, 32.3) | 0.1 |
| ***Comorbidities*** |  |  |  |  |  |  |  |  |
| Dyslipidaemia | 62 | 20 (69%) | 24 (73%) | 0.7 | 68 | 26 (76%) | 24 (71%) | 0.6 |
| Smoking habits | 62 | 7 (24%) | 2 (6.1%) | 0.07 | 68 | 10 (29%) | 2 (5.9%) | **0.011** |
| Arterial hypertension | 62 | 21 (72%) | 27 (82%) | 0.4 | 68 | 27 (79%) | 27 (79%) | >0.9 |
| Diabetes mellitus | 62 | 15 (52%) | 10 (30%) | 0.086 | 68 | 13 (38%) | 11 (32%) | 0.6 |
| CAD | 62 | 8 (28%) | 6 (18%) | 0.4 | 68 | 10 (29%) | 4 (12%) | 0.072 |
| AR (mild-moderate) | 62 | 17 (59%) | 20 (61%) | 0.9 | 68 | 21 (62%) | 21 (62%) | >0.9 |
| ***Pharmacology*** |  |  |  |  |  |  |  |  |
| Statins | 62 | 23 (79%) | 24 (73%) | 0.5 | 67 | 27 (82%) | 22 (65%) | 0.11 |
| Beta-blockers | 62 | 12 (41%) | 12 (36%) | 0.7 | 67 | 12 (36%) | 10 (29%) | 0.5 |
| ACEi | 62 | 8 (28%) | 11 (33%) | 0.6 | 67 | 8 (24%) | 12 (35%) | 0.3 |
| AT2Ri | 62 | 11 (38%) | 15 (45%) | 0.5 | 67 | 14 (42%) | 14 (41%) | >0.9 |
| Diuretics | 62 | 13 (45%) | 20 (61%) | 0.2 | 67 | 15 (45%) | 18 (53%) | 0.5 |
| Anticoagulants | 62 | 2 (6.9%) | 1 (3.0%) | 0.6 | 67 | 1 (3.0%) | 1 (2.9%) | >0.9 |
| Aspirin | 62 | 17 (59%) | 13 (39%) | 0.13 | 67 | 18 (55%) | 11 (32%) | 0.067 |
| CAC blockers | 62 | 11 (38%) | 13 (39%) | >0.9 | 67 | 12 (36%) | 11 (32%) | 0.7 |
| SGLT2i | 62 | 5 (17%) | 1 (3.0%) | 0.089 | 67 | 6 (18%) | 1 (2.9%) | 0.054 |
| ***Disease severity*** |  |  |  |  |  |  |  |  |
| taoGmax (mmHg) | 60 | 72 (65, 82) | 79 (68, 87) | 0.5 | 67 | 74 (66, 85) | 81 (70, 91) | 0.3 |
| taoGmean (mmHg) | 62 | 45 (41, 51) | 47 (41, 53) | 0.5 | 68 | 45 (41, 54) | 51 (44, 60) | 0.2 |
| AVAi (cm^2^/m^2^) | 62 | 0.48 (0.43, 0.53) | 0.46 (0.41, 0.50) | 0.4 | 68 | 0.46 (0.41, 0.52) | 0.45 (0.38, 0.50) | 0.4 |
| DVI | 41 | 0.22 (0.20, 0.25) | 0.24 (0.21, 0.25) | 0.3 | 46 | 0.22 (0.19, 0.25) | 0.23 (0.21, 0.25) | 0.4 |
| LVMi (g/m^2^) | 58 | 121 (110, 139) | 118 (108, 138) | 0.8 | 62 | 126 (109, 143) | 118 (105, 138) | 0.5 |
| Bicuspid valves | 58 | 10 (34%) | 11 (38%) | >0.9 | 57 | 10 (30%) | 13 (45%) | 0.3 |
| ^a^n (%); Median (IQR); ^b^Wilcoxon rank sum test; Pearson's Chi-squared test; Fisher's exact test; Wilcoxon rank sum exact test | | | | | | | | |
| ACEi – angiotensin-converting enzyme inhibitors; AR – aortic regurgitation; AT2Ri – angiotensin II receptor inhibitors; AVAi – aortic valve area indexed to body surface area; AVR – aortic valve replacement; BMI – body mass index; CAC blockers – calcium channel blockers; CAD – coronary artery disease; DVI – Doppler velocity index (velocity ratio); LVMi – left ventricle mass indexed to body surface area; SGLT2i – sodium-glucose co-transporter 2 inhibitors; taoGmax – maximal transvalvular aortic pressure gradient; taoGmean – mean transvalvular aortic pressure gradient | | | | | | | | |

**Supplementary Table 4.** Proteins differentially expressed between men and women with aortic valve stenosis.

| **Protein ID** | **Gene Name** | **Protein Name** | **N (men)** | **N (women)** | **Sequenced peptides** | **FC** | ***p*** | **Adj. *p*** | **Cohen's d** |
| --- | --- | --- | --- | --- | --- | --- | --- | --- | --- |
| P22891 | PROZ | Vitamin K-dependent protein Z | 24 | 24 | 11 | 0.32 | 0.0022 | 0.18 | 0.91 |
| Q9BRR6 | ADPGK | ADP-dependent glucokinase | 25 | 24 | 9 | 0.33 | 0.0030 | 0.19 | 0.87 |
| Q9BZZ2 | SIGLEC1 | Sialoadhesin | 24 | 22 | 31 | 0.50 | 0.0002 | 0.14 | 1.14 |
| Q9NNX6 | CD209 | CD209 antigen | 25 | 20 | 11 | 0.56 | 0.0025 | 0.19 | 0.91 |
| O60496 | DOK2 | Docking protein 2 | 25 | 25 | 10 | 0.56 | 0.0006 | 0.14 | 1.02 |
| P15144 | ANPEP | Aminopeptidase N | 25 | 25 | 39 | 0.57 | 0.0015 | 0.17 | 0.93 |
| Q5JTJ3 | COA6 | Cytochrome c oxidase assembly factor 6 homolog | 18 | 15 | 4 | 0.58 | 0.0024 | 0.19 | 1.10 |
| P55008 | AIF1 | Allograft inflammatory factor 1 | 24 | 24 | 6 | 0.62 | 0.0004 | 0.14 | 1.10 |
| Q687X5 | STEAP4 | Metalloreductase STEAP4 | 25 | 24 | 16 | 0.62 | 0.0008 | 0.14 | 1.01 |
| P53634 | CTSC | Dipeptidyl peptidase 1 | 25 | 25 | 13 | 0.62 | 0.0025 | 0.19 | 0.89 |
| P00488 | F13A1 | Coagulation factor XIII A chain | 25 | 25 | 38 | 0.64 | 0.0003 | 0.14 | 1.10 |
| Q13451 | FKBP5 | Peptidyl-prolyl cis-trans isomerase FKBP5 | 25 | 25 | 18 | 0.65 | 0.0001 | 0.14 | 1.23 |
| O00754 | MAN2B1 | Lysosomal alpha-mannosidase | 24 | 25 | 16 | 0.65 | 0.0038 | 0.20 | 0.87 |
| P50135 | HNMT | Histamine N-methyltransferase | 25 | 25 | 8 | 0.66 | 0.0021 | 0.18 | 0.92 |
| P28838 | LAP3 | Cytosol aminopeptidase | 25 | 25 | 31 | 0.67 | 0.0007 | 0.14 | 1.04 |
| P02743 | APCS | Serum amyloid P-component | 25 | 25 | 14 | 0.67 | 0.0005 | 0.14 | 1.03 |
| P04839 | CYBB | Cytochrome b-245 heavy chain | 25 | 25 | 12 | 0.70 | 0.0036 | 0.20 | 0.85 |
| Q86VB7 | CD163 | Scavenger receptor cysteine-rich type 1 protein M130 | 25 | 25 | 39 | 0.70 | 0.0003 | 0.14 | 1.10 |
| P58335 | ANTXR2 | Anthrax toxin receptor 2 | 25 | 25 | 9 | 0.71 | 0.0006 | 0.14 | 1.05 |
| P29144 | TPP2 | Tripeptidyl-peptidase 2 | 25 | 25 | 35 | 0.72 | 0.0039 | 0.20 | 0.87 |
| Q9Y279 | VSIG4 | V-set and immunoglobulin domain-containing protein 4 | 25 | 25 | 11 | 0.73 | 0.0005 | 0.14 | 1.03 |
| P14317 | HCLS1 | Hematopoietic lineage cell-specific protein | 25 | 25 | 20 | 0.74 | 0.0018 | 0.17 | 0.92 |
| Q53H82 | LACTB2 | Endoribonuclease LACTB2 | 25 | 22 | 10 | 0.74 | 0.0021 | 0.18 | 0.99 |
| Q9NY15 | STAB1 | Stabilin-1 | 25 | 25 | 56 | 0.75 | 0.0011 | 0.14 | 0.98 |
| Q86SX6 | GLRX5 | Glutaredoxin-related protein 5, mitochondrial | 23 | 22 | 3 | 0.75 | 0.0012 | 0.14 | 1.04 |
| P22897 | MRC1 | Macrophage mannose receptor 1 | 25 | 25 | 50 | 0.75 | 0.0032 | 0.19 | 0.88 |
| P30273 | FCER1G | High affinity immunoglobulin epsilon receptor subunit gamma | 25 | 25 | 3 | 0.75 | 0.0038 | 0.20 | 0.84 |
| P98082 | DAB2 | Disabled homolog 2 | 25 | 25 | 19 | 0.75 | 0.0028 | 0.19 | 0.90 |
| Q8WU79 | SMAP2 | Stromal membrane-associated protein 2 | 25 | 24 | 19 | 0.76 | 0.0007 | 0.14 | 1.06 |
| P36269 | GGT5 | Glutathione hydrolase 5 proenzyme | 25 | 25 | 16 | 0.77 | 0.0041 | 0.20 | 0.85 |
| P18859 | ATP5PF | ATP synthase-coupling factor 6, mitochondrial | 25 | 25 | 5 | 0.78 | 0.0023 | 0.19 | 0.93 |
| Q4VC31 | MIX23 | Protein MIX23 | 24 | 22 | 3 | 0.78 | 0.0009 | 0.14 | 1.04 |
| P04233 | CD74 | HLA class II histocompatibility antigen gamma chain | 25 | 25 | 7 | 0.78 | 0.0007 | 0.14 | 1.01 |
| P07203 | GPX1 | Glutathione peroxidase 1 | 25 | 25 | 14 | 0.78 | 0.0015 | 0.17 | 0.98 |
| P52566 | ARHGDIB | Rho GDP-dissociation inhibitor 2 | 25 | 25 | 13 | 0.82 | 0.0031 | 0.19 | 0.90 |
| Q9BS26 | ERP44 | Endoplasmic reticulum resident protein 44 | 25 | 25 | 15 | 0.85 | 0.0018 | 0.17 | 1.02 |
| P09496 | CLTA | Clathrin light chain A | 25 | 25 | 8 | 0.85 | 0.0010 | 0.14 | 1.07 |
| P82909 | MRPS36 | 28S ribosomal protein S36, mitochondrial | 25 | 25 | 3 | 0.85 | 0.0009 | 0.14 | 1.08 |
| Q16698 | DECR1 | 2,4-dienoyl-CoA reductase [(3E)-enoyl-CoA-producing], mitochondrial | 25 | 25 | 15 | 0.86 | 0.0004 | 0.14 | 1.10 |
| P30048 | PRDX3 | Thioredoxin-dependent peroxide reductase, mitochondrial | 25 | 25 | 7 | 0.86 | 0.0010 | 0.14 | 1.05 |
| P50213 | IDH3A | Isocitrate dehydrogenase [NAD] subunit alpha, mitochondrial | 25 | 25 | 14 | 0.89 | 0.0038 | 0.20 | 0.90 |
| P26599 | PTBP1 | Polypyrimidine tract-binding protein 1 | 25 | 25 | 10 | 0.90 | 0.0040 | 0.20 | 0.93 |
| P17858 | PFKL | ATP-dependent 6-phosphofructokinase, liver type | 25 | 25 | 27 | 1.23 | 0.0028 | 0.19 | 0.90 |
| Q9Y6N7 | ROBO1 | Roundabout homolog 1 | 25 | 23 | 21 | 1.30 | 0.0009 | 0.14 | 1.03 |
| Q13642 | FHL1 | Four and a half LIM domains protein 1 | 25 | 25 | 16 | 1.39 | 0.0001 | 0.14 | 1.20 |
| Q08623 | PUDP | Pseudouridine-5'-phosphatase | 20 | 21 | 5 | 1.42 | 0.0030 | 0.19 | 0.98 |
| P12429 | ANXA3 | Annexin A3 | 25 | 25 | 20 | 1.43 | 0.0007 | 0.14 | 1.01 |
| P98095 | FBLN2 | Fibulin-2 | 25 | 25 | 39 | 1.44 | 0.0008 | 0.14 | 1.00 |
| P62917 | RPL8 | 60S ribosomal protein L8 | 20 | 17 | 6 | 1.44 | 0.0026 | 0.19 | 1.04 |
| Q13418 | ILK | Integrin-linked protein kinase | 25 | 25 | 20 | 1.46 | 0.0042 | 0.20 | 0.84 |
| Q13884 | SNTB1 | Beta-1-syntrophin | 24 | 22 | 12 | 1.50 | 0.0030 | 0.19 | 0.92 |
| P23352 | ANOS1 | Anosmin-1 | 25 | 25 | 19 | 1.50 | 0.0017 | 0.17 | 0.92 |
| P50579 | METAP2 | Methionine aminopeptidase 2 | 20 | 21 | 13 | 1.55 | 0.0022 | 0.18 | 0.99 |
| O75131 | CPNE3 | Copine-3 | 25 | 25 | 15 | 1.68 | 0.0032 | 0.19 | 0.87 |
| P62899 | RPL31 | 60S ribosomal protein L31 | 25 | 23 | 4 | 1.70 | 0.0041 | 0.20 | 0.85 |
| P62701 | RPS4X | 40S ribosomal protein S4, X isoform | 25 | 25 | 10 | 1.77 | 0.0038 | 0.20 | 0.85 |
| O95219 | SNX4 | Sorting nexin-4 | 22 | 22 | 15 | 1.80 | 0.0015 | 0.17 | 0.99 |
| Q96BJ3 | AIDA | Axin interactor, dorsalization-associated protein | 22 | 22 | 10 | 1.87 | 0.0003 | 0.14 | 1.15 |
| Q9NRF8 | CTPS2 | CTP synthase 2 | 19 | 20 | 12 | 1.90 | 0.0016 | 0.17 | 1.06 |
| P22059 | OSBP | Oxysterol-binding protein 1 | 23 | 21 | 17 | 1.93 | 0.0036 | 0.20 | 0.90 |
| Q9HCJ6 | VAT1L | Synaptic vesicle membrane protein VAT-1 homolog-like | 24 | 23 | 12 | 1.94 | 0.0008 | 0.14 | 1.03 |
| P09914 | IFIT1 | Interferon-induced protein with tetratricopeptide repeats 1 | 18 | 16 | 14 | 1.94 | 0.0009 | 0.14 | 1.22 |
| Q13442 | PDAP1 | 28 kDa heat- and acid-stable phosphoprotein | 17 | 18 | 5 | 1.94 | 0.0030 | 0.19 | 1.05 |
| Q9Y383 | LUC7L2 | Putative RNA-binding protein Luc7-like 2 | 17 | 18 | 7 | 1.96 | 0.0009 | 0.14 | 1.19 |
| Q9Y2A7 | NCKAP1 | Nck-associated protein 1 | 23 | 24 | 28 | 1.98 | 0.0031 | 0.19 | 0.89 |
| Q9H3P7 | ACBD3 | Golgi resident protein GCP60 | 21 | 20 | 15 | 2.03 | 0.0041 | 0.20 | 0.93 |
| Q99700 | ATXN2 | Ataxin-2 | 19 | 15 | 10 | 2.07 | 0.0031 | 0.19 | 1.11 |
| Q15459 | SF3A1 | Splicing factor 3A subunit 1 | 19 | 18 | 16 | 2.08 | 0.0040 | 0.20 | 0.98 |
| P41159 | LEP | Leptin | 15 | 18 | 4 | 2.09 | 0.0032 | 0.19 | 1.10 |
| P31689 | DNAJA1 | DnaJ homolog subfamily A member 1 | 16 | 18 | 6 | 2.19 | 0.0009 | 0.14 | 1.21 |
| O00186 | STXBP3 | Syntaxin-binding protein 3 | 20 | 22 | 17 | 2.34 | 0.0040 | 0.20 | 0.92 |
| Q9Y2W1 | THRAP3 | Thyroid hormone receptor-associated protein 3 | 21 | 18 | 14 | 2.47 | 0.0023 | 0.19 | 1.00 |
| O14617 | AP3D1 | AP-3 complex subunit delta-1 | 19 | 17 | 19 | 2.81 | 0.0009 | 0.14 | 1.16 |
| Q6P2Q9 | PRPF8 | Pre-mRNA-processing-splicing factor 8 | 19 | 17 | 36 | 2.93 | 0.0022 | 0.19 | 1.06 |
| Q9UNF1 | MAGED2 | Melanoma-associated antigen D2 | 19 | 20 | 14 | 3.03 | 0.0042 | 0.20 | 0.94 |
| Q8IVF7 | FMNL3 | Formin-like protein 3 | 17 | 18 | 15 | 6.69 | 0.0010 | 0.14 | 1.17 |

Adj. p – Adjusted p-value; FC – Fold-change.

**Supplementary Table 5.** Cell type enrichment analysis for men's and women’s proteome based on the Human Gene Atlas.

| **Men** | | | | | | |
| --- | --- | --- | --- | --- | --- | --- |
| Term | Overlap | P-value | Adjusted P-value | Odds Ratio | Combined Score | Genes |
| **CD14+ Monocytes** | 8/383 | 0.000001 | **0.000026** | 12.28737 | **168.3305** | CD163; DOK2; FCER1G; STAB1; CYBB; MAN2B1; HNMT; AIF1 |
| **CD33+ Myeloid** | 7/679 | 0.000483 | **0.005552** | 5.739881 | **43.82926** | CD163; ADPGK; STAB1; CYBB; HCLS1; HNMT; AIF1 |
| **Lymph node** | 2/37 | 0.002737 | **0.020981** | 28.46143 | **167.9512** | CD209; SIGLEC1 |
| BDCA4+ Dendritic Cells | 4/495 | 0.019761 | 0.102763 | 4.173438 | 16.37669 | ADPGK; MAN2B1; LAP3; IDH3A |
| Whole Blood | 4/514 | 0.02234 | 0.102763 | 4.014035 | 15.25891 | STEAP4; ARHGDIB; CYBB; AIF1 |
| Adipocyte | 2/181 | 0.055388 | 0.212321 | 5.52486 | 15.98557 | DECR1; FKBP5 |
| Lung | 2/299 | 0.130081 | 0.42741 | 3.309933 | 6.750927 | GPX1; VSIG4 |
| Placenta | 2/405 | 0.208824 | 0.60037 | 2.426179 | 3.80003 | DAB2; F13A1 |
| Appendix | 1/127 | 0.234959 | 0.60045 | 3.838947 | 5.560124 | CD163 |
| Superior Cervical Ganglion | 2/602 | 0.36196 | 0.713148 | 1.613167 | 1.639333 | CD209; AIF1 |
| Small intestine | 1/220 | 0.371889 | 0.713148 | 2.198352 | 2.174521 | ANPEP |
| Liver | 2/618 | 0.374143 | 0.713148 | 1.569968 | 1.543461 | APCS; ANPEP |
| Pineal gland | 2/694 | 0.430705 | 0.713148 | 1.392052 | 1.17257 | ERP44; LACTB2 |
| Atrioventricular Node | 1/269 | 0.43409 | 0.713148 | 1.791955 | 1.495393 | CLTA |
| Lymphoma Burkitt (Raji) | 1/355 | 0.529036 | 0.756215 | 1.350696 | 0.859987 | CD74 |
| CD105+ Endothelial | 1/370 | 0.54392 | 0.756215 | 1.294798 | 0.78847 | MRPS36 |
| Skeletal Muscle | 1/441 | 0.608368 | 0.756215 | 1.081929 | 0.537692 | HNMT |
| 721 B lymphoblasts | 3/1543 | 0.63895 | 0.756215 | 0.91998 | 0.412086 | PTBP1; LAP3; IDH3A |
| CD4+ T cells | 1/533 | 0.678791 | 0.756215 | 0.890611 | 0.34506 | TPP2 |
| CD71+ Early Erythroid | 1/553 | 0.692377 | 0.756215 | 0.857458 | 0.315223 | GLRX5 |
| Testis Interstitial | 1/568 | 0.702197 | 0.756215 | 0.834129 | 0.294899 | TPP2 |
| CD8+ T cells | 1/602 | 0.723336 | 0.756215 | 0.785561 | 0.254429 | TPP2 |
| CD56+ NK Cells | 1/752 | 0.800379 | 0.800379 | 0.623786 | 0.138899 | DOK2 |
| **Women** | | | | | | |
| Term | Overlap | P-value | Adjusted P-value | Odds Ratio | Combined Score | Genes |
| Leukaemia lymphoblastic (MOLT-4) | 2/118 | 0.017121 | 0.280849 | 10.69504 | 43.50176 | SF3A1; LUC7L2 |
| Thyroid | 3/388 | 0.027806 | 0.280849 | 4.92191 | 17.63274 | SNX4; STXBP3; FHL1 |
| CD19+ B Cells (neg. sel.) | 3/412 | 0.032406 | 0.280849 | 4.627415 | 15.86936 | SF3A1; AIDA; ACBD3 |
| Fetal brain | 2/325 | 0.105213 | 0.527569 | 3.80089 | 8.558718 | DNAJA1; ROBO1 |
| Fetal Thyroid | 1/66 | 0.106383 | 0.527569 | 9.277855 | 20.78897 | RPS4X |
| Lymphoma Burkitt (Raji) | 2/355 | 0.121747 | 0.527569 | 3.472557 | 7.312555 | PFKL; RPL8 |
| Placenta | 2/405 | 0.150594 | 0.534017 | 3.033964 | 5.743812 | LEP; OSBP |
| Tongue | 1/115 | 0.178174 | 0.534017 | 5.27698 | 9.102774 | FHL1 |
| Appendix | 1/127 | 0.194883 | 0.534017 | 4.771525 | 7.803148 | NCKAP1 |
| BDCA4+ Dendritic Cells | 2/495 | 0.205391 | 0.534017 | 2.468687 | 3.907534 | AP3D1; CPNE3 |
| Adipocyte | 1/181 | 0.266081 | 0.628919 | 3.330976 | 4.410061 | FBLN2 |
| Small intestine | 1/220 | 0.313661 | 0.639855 | 2.732392 | 3.168051 | CPNE3 |
| Pineal gland | 2/694 | 0.331233 | 0.639855 | 1.740788 | 1.923451 | OSBP; PRPF8 |
| Prostate | 1/249 | 0.347086 | 0.639855 | 2.409335 | 2.549515 | MAGED2 |
| Bronchial Epithelial Cells | 1/280 | 0.381069 | 0.639855 | 2.138264 | 2.062945 | ANXA3 |
| Colorectal adenocarcinoma | 1/292 | 0.393757 | 0.639855 | 2.048839 | 1.909561 | PDAP1 |
| Amygdala | 1/345 | 0.446841 | 0.675612 | 1.728506 | 1.392403 | NCKAP1 |
| CD105+ Endothelial | 1/370 | 0.470287 | 0.675612 | 1.609345 | 1.214111 | METAP2 |
| 721 B lymphoblasts | 3/1543 | 0.493716 | 0.675612 | 1.157897 | 0.817237 | SNX4; PFKL; CTPS2 |
| Skeletal Muscle | 1/441 | 0.531735 | 0.691255 | 1.344766 | 0.849368 | FHL1 |
| Whole Blood | 1/514 | 0.587683 | 0.695055 | 1.149093 | 0.61082 | IFIT1 |
| Prefrontal Cortex | 1/521 | 0.592694 | 0.695055 | 1.133217 | 0.59276 | ATXN2 |
| CD71+ Early Erythroid | 1/553 | 0.614856 | 0.695055 | 1.065766 | 0.518354 | METAP2 |
| Superior Cervical Ganglion | 1/602 | 0.646541 | 0.69914 | 0.976403 | 0.425828 | SNTB1 |
| CD34+ | 1/645 | 0.67225 | 0.69914 | 0.909185 | 0.36106 | RPL31 |
| CD56+ NK Cells | 1/752 | 0.728596 | 0.728596 | 0.77533 | 0.245497 | AP3D1 |

**Supplementary Table 6.** Selection of proteins for validation.

| **Gene name** | **Protein Name** | **Regulation** | **Justification** |
| --- | --- | --- | --- |
| AIF1 | Allograft inflammatory factor 1 | ♂>♀ | Calcium-binding protein playing a role in several immune-inflammatory processes, including macrophage activation, phagocytosis, lymphocyte migration and proliferation.  Near-central position in the PPI network. |
| ANPEP | Aminopeptidase N | ♂>♀ | Involved in the processing of chemokines and peptides bound to MHC class II in antigen-presenting cells.  May promote cholesterol crystallisation.  Correlates with valve weight (irrespective of sex). |
| CD163 | Scavenger receptor cysteine-rich type 1 protein M130 | ♂>♀ | Marker of monocytes/macrophages.  Involved in haemoglobin/haptoglobin complexes clearance, a source of oxidative stress in the AV.  Hub protein in the PPI network (largest degree: 11).  Correlates with AVA (irrespective of sex). |
| CD74 | HLA class II histocompatibility antigen gamma chain | ♂>♀ | Marker of macrophages, dendritic and B cells.  Plays a critical role in MHC class II antigen processing.  Near-central position in the PPI network.  Correlates with DVI (irrespective of sex). |
| DNAJA1 | DnaJ homolog subfamily A member 1 | ♂<♀ | Co-chaperone that protects cells from apoptosis in response to cell stress by inhibiting translocation of Bax to mitochondria.  Inversely correlates with valve weight (irrespective of sex). |
| F13A1 | Coagulation factor XIII A chain | ♂>♀ | Participates in blood coagulation by stabilising fibrin clots.  F13A1 may promote foam cell formation in atherosclerotic lesions.  Near-central position in the PPI network.  Correlates with LV hypertrophy (only in women). |
| GPX1 | Glutathione peroxidase 1 | ♂>♀ | Relevant oxidative stress protective enzyme.  Major bottleneck in the PPI network (betweenness centrality: 0.35). |
| CYBB/ NOX2 | Cytochrome b-245 heavy chain/NADPH oxidase 2 | ♂>♀ | Critical component of the membrane-bound oxidase of phagocytes that generates superoxide.  Central-most protein in the PPI network (betweenness centrality: 0.39), also behaves as a hub (node degree:10).  Strongly correlates with DVI in women. |
| OSBP1 | Oxysterol-binding protein 1 | ♂<♀ | Lipid transporter that regulates cholesterol efflux.  Inversely correlates with DVI in women, correlates with AVAi in men. |
| PFKL | ATP-dependent 6-phosphofructokinase, liver type | ♂<♀ | Negatively regulates the phagocyte oxidative burst by controlling cellular NADPH biosynthesis and NADPH oxidase-derived reactive oxygen species.  Negatively correlates with valve weight (irrespective of sex). |
| STEAP4 | Metalloreductase STEAP4 | ♂>♀ | Responsible for the reduction of Fe^3+^-chelates.  Important for systemic metabolic homeostasis, integrating inflammatory and metabolic responses.  Strongly correlates with valve weight (irrespective of sex). |

Abbreviations: AVA: aortic valve area; AVAi: AVA, indexed to body surface area; DVI: Doppler velocity index (velocity ratio); LV: left ventricle; PPI: protein-protein interaction.

**Supplementary Table 7.** Significant correlations between transcript levels and disease severity in men and women.

| **Sex** | **Men** | | | | | **Women** | | | | |
| --- | --- | --- | --- | --- | --- | --- | --- | --- | --- | --- |
| **Genes** | taoG  mean | AVA | DVI | AVW | LVM | taoG  mean | AVA | DVI | AVW | LVM |
| **AIF1** |  |  |  |  |  |  | r=0.44  *p*=0.036 |  |  |  |
| **ANPEP** | r=-0.44  *p*=0.035 |  |  |  |  |  |  |  |  |  |
| **CD163** |  |  |  |  |  |  |  |  |  |  |
| **CD74** |  |  |  |  | r =0.45  *p*=0.033 |  |  |  |  |  |
| **DNAJA1** | r=-0.56  *p*=0.0045 |  |  |  |  |  | r=0.60  *p*=0.0030 |  |  |  |
| **F13A1** |  |  |  | r=0.59  *p*=0.0032 |  |  |  |  |  |  |
| **GPX1** |  |  |  |  |  |  |  |  |  |  |
| **NOX2** |  |  |  |  |  |  |  |  |  |  |
| **OSBP** |  |  |  |  |  |  |  |  |  |  |
| **PFKL** |  |  |  | r=-0.52  *p*=0.016 | r=-0.53  *p*=0.015 |  |  |  |  |  |
| **STEAP4** | r=-0.48  *p*=0.018 |  |  |  |  |  |  |  |  |  |
| **TGFB** |  |  |  |  |  |  |  |  |  |  |
| **COL1A1** |  |  |  |  |  |  |  |  |  |  |
| **COL3A1** |  |  |  |  |  |  |  |  |  |  |
| **MMP2** |  |  |  |  |  |  |  |  |  |  |
| **MMP9** |  |  | r=-0.62  *p*=0.012 |  |  |  |  |  |  |  |
| **TIMP1** |  |  |  |  |  |  |  |  |  |  |
| **TIMP2** |  |  |  |  |  |  |  |  |  |  |

Abbreviations: AVA: aortic valve area; AVW: aortic valve weight; DVI: Doppler velocity index (velocity ratio); LVM: left ventricle mass; taoGmean: mean transvalvular aortic pressure gradient.

**Supplementary Table 8.** Cell type enrichment analysis for women’s proteome based on the Human Gene Atlas (considering all significantly dysregulated proteins, no p-value adjustment)

| Term | Overlap | P-value | Adjusted P-value | Odds Ratio | Combined Score | Genes |
| --- | --- | --- | --- | --- | --- | --- |
| **Smooth Muscle** | 15/363 | 5.81E-04 | **0.037758** | 2.826125 | 21.05727 | MYOF; LRRC17; RRAS2; PARVA; SGCE; EHD2; MYO1B; NFIB; RPL38; PTGFRN; TMSB10; MYH10; FERMT2; EIF4B; CBR3 |
| 721 B lymphoblasts | 38/1543 | 0.003088 | 0.068991 | 1.688074 | 9.757656 | SF3B4; UNC119B; SF3B5; SET; HSP90AB1; PDCD5; HMGB1; NAPG; RIC8A; SNX4; CAND1; SNRPD1; PPP1R7; PMVK; TMPO; KPNA1; CCT3; SMAD2; USP9X; EIF1AX; PTGES3; CTPS2; NAP1L1; PA2G4; SRP68; CKAP5; NAALADL2; NIPSNAP1; ILF3; PFKL; SNRNP40; MYO1C; PLSCR3; PSAT1; SUB1; EXOC4; DRG2; SNRPE |
| Fetal Thyroid | 5/66 | 0.003578 | 0.068991 | 5.275195 | 29.71533 | RPS4X; CAMK2D; SNX1; SDC2; RPL38 |
| Uterus | 9/201 | 0.004246 | 0.068991 | 3.036441 | 16.58464 | SVIL; CUL5; PALLD; PARVA; MYH11; PKD2; MYL9; FERMT2; STRN3 |
| Adipocyte | 8/181 | 0.007397 | 0.096166 | 2.988478 | 14.66334 | EHD2; PALLD; PARVA; COPS8; FBLN2; FBLN5; FERMT2; PHLDA3 |
| Olfactory Bulb | 4/83 | 0.040102 | 0.374351 | 3.244974 | 10.43686 | SGCD; KIF13B; PHLDA3; LHPP |
| Thyroid | 11/388 | 0.040315 | 0.374351 | 1.884646 | 6.05167 | UNC119B; SNX4; FNDC1; STXBP3; GPX3; SDC2; DCAKD; FHL1; LTBP4; MAGT1; MATN2 |
| Bronchial Epithelial Cells | 8/280 | 0.071036 | 0.542232 | 1.891118 | 5.001183 | EHD2; MYO1B; ANXA3; MYOF; RRAS2; ITGA6; ACSL3; BZW1 |
| Spinal cord | 4/105 | 0.080618 | 0.542232 | 2.535301 | 6.383976 | AHCYL1; TBC1D9B; SAP18; LHPP |
| BDCA4+ Dendritic Cells | 12/495 | 0.08547 | 0.542232 | 1.601317 | 3.938593 | DNAJC3; CYFIP1; GIT2; TFCP2; MICAL1; AP3D1; CPNE3; SNX9; PPIB; EXOC2; CORO1C; DNM2 |
| Dorsal Root Ganglion | 3/69 | 0.091762 | 0.542232 | 2.905537 | 6.940029 | PURB; PRKAB2; SLC2A13 |
| Leukemia lymphoblastic (MOLT-4) | 4/118 | 0.111332 | 0.603049 | 2.244697 | 4.927643 | SF3A1; CBX1; CPNE1; LUC7L2 |
| CD71+ Early Erythroid | 12/553 | 0.152844 | 0.726776 | 1.425325 | 2.67724 | CUL4A; EIF5; NFIX; USP9X; ENDOD1; PIP4K2A; AP2A1; HDGF; AP2B1; SPTB; TMPO; METAP2 |
| Colorectal adenocarcinoma | 7/292 | 0.169376 | 0.726776 | 1.572984 | 2.793047 | ABLIM1; STK24; CTNNA1; PDAP1; ITGA6; IPO7; LIMS1 |
| Prefrontal Cortex | 11/521 | 0.187539 | 0.726776 | 1.383566 | 2.315766 | CLIP2; ATXN2; AHCYL1; EIF1AX; ROCK2; FKBP8; ANK3; ACSL3; DNM1; GAS7; CAP2 |
| Lymphoma burkitts (Raji) | 8/355 | 0.187684 | 0.726776 | 1.476649 | 2.47043 | EHD1; PFKL; NEK6; NAP1L1; PKN1; RPL8; IPO7; NIPSNAP1 |
| CD56+ NK Cells | 15/752 | 0.192335 | 0.726776 | 1.307614 | 2.155625 | SMAD2; PRKAG1; AP3D1; CACNA2D2; MSN; RIC8A; EHD1; DNAJC3; ANXA6; SAP18; ESYT1; OSTF1; GBP2; EXOC2; JAK1 |
| CD34+ | 13/645 | 0.203548 | 0.726776 | 1.319919 | 2.101117 | SET; RPL31; RPL22; NAP1L1; HMGB1; ILF3; COPS5; CAND1; SNRPD1; NACA; TPR; EXOC4; KPNA1 |
| colon | 6/258 | 0.212442 | 0.726776 | 1.5224 | 2.358326 | SVIL; PLSCR1; CTNND1; MYH11; WASL; SLC25A24 |
| Globus Pallidus | 2/59 | 0.232518 | 0.745985 | 2.236614 | 3.262749 | PURB; TPPP |
| Fetal brain | 7/325 | 0.241011 | 0.745985 | 1.407352 | 2.002541 | DNAJA1; DAAM1; EXOC4; CRMP1; EML1; DCLK1; ROBO1 |
| Liver | 12/618 | 0.252811 | 0.74694 | 1.268124 | 1.743816 | NUP214; SLC25A1; PRKAB2; ITIH3; C6; RARRES2; PZP; CTNNA1; CDC42EP1; PPIB; LLGL1; CES1 |
| Trachea | 2/72 | 0.307216 | 0.777726 | 1.820037 | 2.148013 | ALDH3A1; CBR3 |
| Heart | 8/415 | 0.315842 | 0.777726 | 1.255056 | 1.44647 | SF3B5; SLC12A4; MACROD1; SCRIB; PPP2R5D; MYL9; USE1; SLC25A4 |
| Whole brain | 5/240 | 0.315864 | 0.777726 | 1.357168 | 1.564058 | VPS18; EHD3; HSPBP1; SLC25A4; CAP2 |
| Lung | 6/299 | 0.319195 | 0.777726 | 1.306606 | 1.492082 | SLC12A4; EHD4; LTBP4; CACNA2D2; MYH9; ENG |
| Parietal Lobe | 1/26 | 0.333964 | 0.777726 | 2.545631 | 2.791852 | PIP4K2A |
| Prostate | 5/249 | 0.343322 | 0.777726 | 1.306504 | 1.396765 | NFIB; MAGED2; MYH11; GPD1L; NAALADL2 |
| Thalamus | 2/79 | 0.346986 | 0.777726 | 1.653989 | 1.750701 | PPP2R5D; HSPA12A |
| Testis Germ Cell | 6/314 | 0.360317 | 0.780686 | 1.242011 | 1.26781 | ISYNA1; HMGN5; DDX3X; TPR; DRG1; AP2B1 |
| Skeletal Muscle | 8/441 | 0.376286 | 0.788988 | 1.178104 | 1.151485 | MYO1B; SGCD; FHL1; DNAJA2; HSPA12A; SPTB; SLC25A4; EIF4G2 |
| Cardiac Myocytes | 5/273 | 0.416928 | 0.824309 | 1.188035 | 1.039341 | NFIB; TPR; NEK7; COPS8; ENG |
| small intestine | 4/220 | 0.445004 | 0.824309 | 1.178528 | 0.954221 | CTNND1; CPNE3; MXRA5; SLC25A24 |
| Amygdala | 6/345 | 0.445749 | 0.824309 | 1.12663 | 0.910317 | NCKAP1; HSP90AA1; CAMK2D; SUB1; KLC1; DCLK1 |
| Uterus Corpus | 2/98 | 0.450275 | 0.824309 | 1.325352 | 1.057494 | CTNNA1; FBLN1 |
| CD19+ B Cells (neg. sel.) | 7/412 | 0.456541 | 0.824309 | 1.100069 | 0.86254 | RPS26; GIT2; SF3A1; ABLIM1; AIDA; EZR; ACBD3 |
| Fetal lung | 1/41 | 0.473299 | 0.831471 | 1.589806 | 1.18922 | ELN |
| pineal day | 11/694 | 0.513131 | 0.877723 | 1.023798 | 0.683104 | MAP1LC3A; TFCP2; CAND1; ST13; OSBP; COPS7A; FNDC3A; TPD52L1; PRPF8; PFKM; MATN2 |
| Whole Blood | 8/514 | 0.545234 | 0.886903 | 1.004319 | 0.60916 | DNAJC3; VPS4B; GMPR2; IFIT5; OSTF1; IFIT1; GBP2; GBP1 |
| CD14+ Monocytes | 6/383 | 0.546712 | 0.886903 | 1.011081 | 0.610524 | NUP214; VPS18; PLSCR1; VCAN; TFCP2; GAS7 |
| Fetal liver | 2/126 | 0.584012 | 0.886903 | 1.024612 | 0.551072 | DAAM1; NAALADL2 |
| Appendix | 2/127 | 0.588321 | 0.886903 | 1.016364 | 0.539163 | NCKAP1; MYH11 |
| Placenta | 6/405 | 0.601312 | 0.886903 | 0.954244 | 0.485368 | TPPP3; LEP; OSBP; FBLN1; EZR; ENG |
| Leukemia promyelocytic-HL-60 | 1/62 | 0.620923 | 0.886903 | 1.041382 | 0.496269 | NIPSNAP1 |
| Cerebellum Peduncles | 2/137 | 0.629593 | 0.886903 | 0.940596 | 0.435197 | ERC1; GAS7 |
| Lymphoma burkitts (Daudi) | 2/139 | 0.637451 | 0.886903 | 0.92677 | 0.417304 | SET; EIF4B |
| Kidney | 2/142 | 0.648993 | 0.886903 | 0.906772 | 0.392028 | GPX3; PARP14 |
| Thymus | 1/68 | 0.654944 | 0.886903 | 0.947834 | 0.401128 | LETM1 |
| Subthalamic Nucleus | 1/77 | 0.700354 | 0.895715 | 0.835207 | 0.297475 | PURB |
| Superior Cervical Ganglion | 8/602 | 0.719247 | 0.895715 | 0.851607 | 0.280648 | GNA14; MYO1B; HMGN5; PRG4; FBLN1; RSU1; NAPG; SNTB1 |
| CD33+ Myeloid | 9/679 | 0.729401 | 0.895715 | 0.848812 | 0.267827 | GIT2; PLSCR1; TMX4; VCAN; TBC1D9B; EIF5; DDX3X; PDCD6IP; GAS7 |
| Pituitary | 1/84 | 0.731511 | 0.895715 | 0.764495 | 0.239014 | CUL5 |
| Hypothalamus | 1/97 | 0.781058 | 0.895715 | 0.660531 | 0.163222 | ENDOD1 |
| Atrioventricular Node | 3/269 | 0.78993 | 0.895715 | 0.713575 | 0.168269 | GNA14; PA2G4; PRKG1 |
| Ciliary Ganglion | 3/278 | 0.808021 | 0.895715 | 0.689902 | 0.147065 | PALLD; NAALADL2; PRKG1 |
| Trigeminal Ganglion | 2/196 | 0.810217 | 0.895715 | 0.652564 | 0.137334 | FNDC3A; RSU1 |
| Cerebellum | 1/108 | 0.815788 | 0.895715 | 0.592294 | 0.120591 | GAS7 |
| pineal night | 8/674 | 0.824073 | 0.895715 | 0.756677 | 0.146414 | TMX4; TFCP2; ST13; PIP4K2C; FNDC3A; TPD52L1; PFKM; MATN2 |
| CD105+ Endothelial | 4/370 | 0.828275 | 0.895715 | 0.690168 | 0.130035 | COPS2; EIF3J; KPNA1; METAP2 |
| Tongue | 1/115 | 0.834971 | 0.895715 | 0.555726 | 0.10023 | FHL1 |
| Testis | 4/384 | 0.849153 | 0.895715 | 0.664259 | 0.108617 | EHD1; TBL2; DRG1; THBS3 |
| retina | 2/218 | 0.854375 | 0.895715 | 0.585438 | 0.092139 | PLSCR4; PI16 |
| Testis Intersitial | 6/568 | 0.877417 | 0.905271 | 0.671755 | 0.087847 | AKAP12; PPP1R7; HSPA4L; DRG1; AP2B1; KPNA1 |
| CD8+ T cells | 6/602 | 0.908135 | 0.922325 | 0.632307 | 0.060931 | ABLIM1; NACA; RPL22; ESYT1; PARP14; GBP1 |
| CD4+ T cells | 4/533 | 0.967174 | 0.967174 | 0.473479 | 0.015803 | PLSCR3; ESYT1; GBP1; EIF4B |

**Supplementary Files**

**Supplementary File 1.** Raw protein quantification data

**Supplementary File 2.** GPX1 and NOX2 protein and peptide quantification data and annotated spectra

**Supplementary File 3.** List of all significant correlations between proteins and parameters of disease severity in men

**Supplementary File 4.** List of all significant correlations between proteins and parameters of disease severity in women
